# Current Analytical Strategies for mRNA-Based Therapeutics

**DOI:** 10.3390/molecules30071629

**Published:** 2025-04-06

**Authors:** Julien Camperi, Kamalakar Chatla, Emily Freund, Carolina Galan, Steffen Lippold, Axel Guilbaud

**Affiliations:** 1 Cell Therapy Engineering and Development, Genentech, 1 DNA Way, South San Francisco, CA 94080, USA; chatlak@gene.com; 2 Department of Molecular Biology, Genentech, 1 DNA Way, South San Francisco, CA 94080, USA; freunde1@gene.com (E.F.); galanc@gene.com (C.G.); 3 Protein Analytical Chemistry, Genentech, 1 DNA Way, South San Francisco, CA 94080, USA; lippolds@gene.com

**Keywords:** messenger RNA, quality attributes, electrophoresis, chromatography, mass spectrometry, sequencing, functionality

## Abstract

Recent advancements in mRNA technology, utilized in vaccines, immunotherapies, protein replacement therapies, and genome editing, have emerged as promising and increasingly viable treatments. The rapid, potent, and transient properties of mRNA-encoded proteins make them attractive tools for the effective treatment of a variety of conditions, ranging from infectious diseases to cancer and single-gene disorders. The capability for rapid and large-scale production of mRNA therapeutics fueled the global response to the COVID-19 pandemic. For effective clinical implementation, it is crucial to deeply characterize and control important mRNA attributes such as purity/integrity, identity, structural quality features, and functionality. This implies the use of powerful and advanced analytical techniques for quality control and characterization of mRNA. Improvements in analytical techniques such as electrophoresis, chromatography, mass spectrometry, sequencing, and functionality assessments have significantly enhanced the quality and detail of information available for product and process characterization, as well as for routine stability and release testing. Here, we review the latest advancements in analytical techniques for the characterization of mRNA-based therapeutics, typically employed by the biopharmaceutical industry for eventual market release.

## 1. Introduction

In vitro transcribed (IVT) messenger RNA-based therapeutics have emerged as an important class of drugs for the treatment of various diseases, including cancer, cardiovascular diseases, infectious diseases, rare genetic disorders, and autoimmune conditions [1]. They come in many forms including mRNA vaccines, cancer immunotherapies, protein replacement therapies, personalized medicine approaches, and gene-editing tools such as CRISPR. Another advantage is that IVT mRNA offers a versatile platform for rapid development and production, allowing for targeted and innovative treatments across a broad range of medical applications. Unlike protein-based drugs, mRNA-based therapies rely on the body’s cellular machinery to translate the encoded information into therapeutic proteins, allowing rapid development. The most notable success story has been the mRNA vaccines developed in response to the COVID-19 pandemic, which demonstrated the platform’s versatility and scalability. The ability of mRNA to produce almost any protein in the body provides countless opportunities for therapeutic interventions, from vaccines to gene therapies.

As of now, there are more than 100 different approved gene, cell, and RNA therapies over the world, and over 3700 are in various stages of clinical trials, highlighting the rapid expansion and extensive applications of these novel therapies and offering new treatment paradigms where traditional approaches have proven insufficient [2]. As research and clinical advancements progress, the range of cell and gene therapies will continue to grow, enhancing patient outcomes and providing effective treatments. Simultaneously, it is crucial to study and mitigate potential adverse effects associated with genetic modification technologies, particularly those involving chemically modified, long-lasting mRNA therapies that produce altered proteins and peptides [3].

The advantages of mRNA-based therapeutics include a short development timeline, the ability to produce a wide range of proteins, and the inherent adaptability of mRNA-based platforms. Within the scope of mRNA-based therapeutics, several types of RNA are being explored, each with unique properties and benefits. Linearized mRNA is the conventional form used in many mRNA-based therapies with a size that can drastically vary (300–1500 kDa) [4]. It comprises a single, linear strand of nucleotides with a sugar-phosphate backbone encoding the desired protein. The mRNA is typically stabilized by a 5′ cap—a modified guanine nucleotide—which enhances ribosome binding and translation initiation, and a poly(A) tail—a string of adenine nucleotides added to the 3′ end—also improving mRNA stability and preventing degradation by exonucleases. The coding region representing the main part of the mRNA contains the genetic code for the protein sequence, and the read codon is flanked by untranslated regions (5′ and 3′ UTRs) modulating mRNA stability and translation efficiency. Despite its well-characterized nature, linearized mRNA is susceptible to rapid degradation by RNases present in the extracellular environment and within cells. Researchers have addressed these challenges through various chemical modifications, such as incorporating modified nucleosides (e.g., pseudouridine, Ψ; 5-methylcytidine, 5mC) in order to create carbon–carbon bonds instead of nitrogen–carbon bonds, making mRNA more stable [5]. Studies have shown that these modifications can significantly enhance the stability and translation efficiency of linearized mRNA [6].

**Figure 1:**
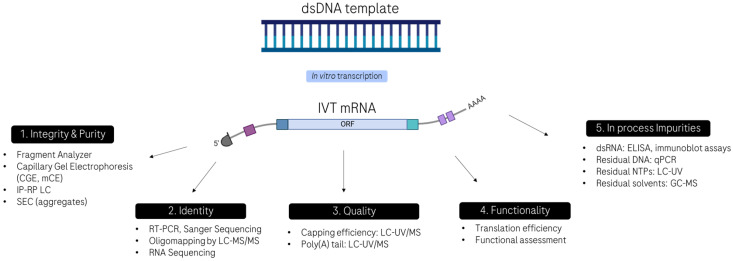
Conventional analytical methods for characterizing the quality attributes of IVT mRNA.

Despite these advantages, producing reproducible, high-quality mRNA-based drugs is a challenge, and developing an analytical characterization package that will meet the expectations of regulatory agencies for a well-characterized product presents several difficulties. mRNA is large, negatively charged, single stranded, highly sensitive to degradation by nucleases, prone to structural heterogeneity, and subject to a variety of chemical modifications, even if efforts are made to improve its stability. In addition to the evaluation of quality attributes tested with traditional therapeutic modalities (i.e., appearance, pH, osmolality, endotoxin levels, bioburden, and sterility), mRNA possesses unique attributes that need to be fully characterized such as its integrity (i.e., full-length mRNA), identity (i.e., sequence confirmation), quality (i.e., capping efficiency and poly(A) tail elements to ensure appropriate stability and protein translation efficiency in vivo), impurities (i.e., double-stranded RNA (dsRNA) for its potential to activate an innate immune response), and functionality (i.e., in vitro translation and cell-based assays), as depicted in Figure 1. Therefore, a comprehensive analytical strategy is essential to ensure the safety, efficacy, and consistency of mRNA products throughout development and manufacturing.

Generally speaking, mRNA medicines must possess a sufficient level of purity, as underscored by documents leaked from the European Medicines Agency that expressed concerns over unexpectedly low quantities (>55%) of intact mRNA in batches of the vaccine developed for commercial production [7]. To ensure the integrity and purity of mRNA therapeutics, tools like the fragment bioanalyzer and capillary gel electrophoresis (CGE) are used to assess RNA lengths and size distribution. In parallel, ion-pair reversed-phase liquid chromatography (IP-RP LC) further aids in the separation of mRNA from impurities based on hydrophobic interactions, while size exclusion chromatography (SEC) can help in identifying aggregates based on size separation.

For identity, various molecular biology techniques are employed. Reverse transcription polymerase chain reaction (RT-PCR) - Sanger Sequencing is the conventional method used to confirm the open reading frame sequence. Oligonucleotide mapping by liquid chromatography-tandem mass spectrometry (LC-MS/MS) and direct RNA sequencing provide detailed information on sequence and chemical modifications. These methods, while powerful, can be time consuming and require high levels of technical expertise and sophisticated instrumentation.

Quality attributes such as capping efficiency and poly(A) tail length are commonly assessed using high-performance liquid chromatography (HPLC) coupled with either ultraviolet (UV) or mass spectrometry (MS) detection [8,9]. Proper capping is essential for efficient translation, and accurate measurement can be challenging due to the structural complexity of the mRNA cap. The poly(A) tail is a sequence of adenine nucleotides added to the end of an mRNA molecule, playing a crucial role in both RNA stability and translation efficiency by facilitating ribosome binding, and its length is often analyzed to understand gene expression regulation as longer tails generally correlate with increased mRNA stability and translation potential [10,11].

Impurities can arise at any step of the manufacturing process and must be carefully monitored to ensure the final product’s purity and safety. In-process impurities such as dsRNA, residual DNA, nucleoside triphosphates (NTPs), and solvents required different analytical assays [12,13,14,15]. Double-strand RNA can elicit strong immune responses and is typically detected using gel electrophoresis or enzyme-linked immunosorbent assays (ELISA) [16,17]. Residual DNA from the production process is quantified using qPCR [18], while residual NTPs [19] and solvents [20] are measured by liquid chromatography and gas chromatography, respectively.

Finally, it is also important to evaluate if the mRNA, after being transfected in specific in vitro models, can be efficiently translated into its corresponding full functional protein. In vitro translation assays and Western blotting are employed to confirm the production of the target protein [21,22,23,24]. Cell-based assays further assess the biological activity of the mRNA product in a relevant cellular context [24,25,26]. These assays are essential for demonstrating therapeutic efficacy but can be limited by variability in cell lines and experimental conditions.

Ensuring the production of high-quality, reproducible IVT mRNA and its quality control are pivotal for the success of therapeutic mRNA applications as the purity of mRNA directly impacts the protein translation efficiency and overall therapeutic efficacy. Impurities such as abortive transcripts, mRNA with a partial or lost poly(A) tail, or aggregates can significantly reduce protein expression, thereby diminishing therapeutic outcomes. Furthermore, impurities like double-stranded RNA (dsRNA) must be meticulously removed during the IVT process to mitigate the immunogenicity of the final mRNA product. Controlling the inherent instability of mRNA molecules under long-term storage and transport conditions is crucial to maintaining the correct functionality of the mRNA throughout its lifecycle. Despite optimizations, IVT mRNA preparations still contain undesired impurities, highlighting the need for sensitive and detailed analytical strategies to measure, control, and understand their relative contributions to biological outcomes. This underscores the importance of characterizing IVT mRNA impurities and understanding their interactions with cellular machinery to ensure effective and safe therapeutic applications.

This review offers a comprehensive overview of the latest advancements in electrophoresis, chromatography, mass spectrometry, sequencing, and functionality assessments used to thoroughly characterize mRNAs. These analytical tools are critical in assessing mRNA sequence integrity (5′capping, 3′poly(A) tail, open reading frame), chemical modifications, and structural heterogeneity, as well as impurities (e.g., abortive transcripts, dsRNA, and aggregates). All these current and innovative technologies play pivotal roles in ensuring the quality, safety, and efficacy of mRNA-based therapeutics. As the field of mRNA therapy continues to grow, these analytical techniques will remain at the forefront of quality control and product development.

## 2. Electrophoretic Approaches

Because of its high resolution and miniaturized format, capillary electrophoresis (CE) is a powerful tool used for the analysis of nucleic acids and especially mRNA based on their size-to-charge ratio. In CE, an electric field is applied across a narrow capillary filled with a conductive buffer. Negatively charged molecules, such as mRNA, migrate through the capillary at different rates depending on their size, their charge, and the viscosity of the buffer. The small internal diameter of the capillary allows for efficient heat dissipation, leading to high resolution and rapid analysis. As mRNA molecules travel through the capillary, they are detected by various means, such as UV absorbance or fluorescence, enabling precise quantification and characterization of the mRNA species. This technique is valued for its high efficiency, speed, and minimal sample requirements, making it a suitable choice for mRNA analysis in research and diagnostic applications.

The characterization and quality control of mRNA are crucial for ensuring therapeutic efficacy, with electrophoresis techniques—particularly CGE and agarose gel electrophoresis (AGE)—playing a central role in these processes [27]. For mRNA therapeutics to be effective, it is essential that the mRNA used in vaccines and therapies is of high integrity and free from degradation products or impurities. Electrophoresis techniques such as CGE are commonly employed to assess these quality and safety attributes, offering a high-resolution method for evaluating full-length linear and circular mRNA and identifying truncated species or degradation products that may have arisen during the manufacturing process, purification, or storage. This becomes especially relevant for mRNA-based vaccines, like those developed against COVID-19, where even minimal levels of degradation can compromise the vaccine’s potency and stability.

During CGE, impurities such as truncated and degradation products are separated from the full-length IVT mRNA transcript. This separation technique relies on the differential migration of analytes—in this context, negatively charged mRNA particles—based on their hydrodynamic radius (size) through a submillimeter-diameter capillary (from approximately 20 to 70 cm of length) filled with a sieving gel buffer solution under the influence of an electric field The different size species are usually detected utilizing a detection mode such as laser-induced fluorescence (LIF) and UV absorption. For instance, LIF, noted for its higher sensitivity, is frequently employed in assays where the quantification and detection of residual impurities are critical as seen in the effort to determine the mRNA purity and integrity of lipid nanoparticle vaccines [28] and detailed poly(A) tail characterization [29]. Table 1 lists most of the CE-based applications for the analysis of mRNA and circRNA.

Since the 1980s, CGE methods have been extensively utilized for the quantitative analysis of mRNA size therapeutics up to 6500 nucleotides (nts) in length, facilitating the separation of small, truncated impurities and aggregates from the full length of the linear IVT mRNA transcript and more recently on circular mRNA (circRNA) [38,39]. Buffer compositions play a pivotal role in optimizing separation efficiency and resolution to be able to study a wide range of mRNA size (e.g., eGFP, FLuc, β-Gal, and Cas9, with sizes of 996, 1909, 3420, and 4500 nts, respectively). The use of standard buffers like Tris-borate-EDTA (TBE) and Tris-acetate-EDTA (TAE) is prevalent due to their consistent pH and ionic strength, which are essential for high-fidelity separations within reasonable timescales, typically 10–40 min (see Table 1).

The work of Lu et al. on non-aqueous CE further demonstrates the flexibility of buffer systems by using formamide to improve the resolution of large RNA molecules, which aqueous buffers might struggle to separate effectively [27]. This approach complements the findings of other studies, who used low-viscous sieving media employing glycerol, polyethylene glycol (PEG), and polyethylene oxide (PEO) in phosphate buffers to balance low viscosity with effective sieving [31,32]. Similarly, the development of dynamic coating buffers, such as hydroxyethyl cellulose (HPC), minimizes capillary wall interactions and accelerates separation times to under 10 min, crucial for high-throughput applications [35]. Expanding on buffer versatility, studies have incorporated various concentration ranges of denaturing agents such as formamide or urea to denature secondary RNA structures [29,33,34,40], methylcellulose to modify the viscosity of the electrophoretic medium, and HEPES combined with polymer solutions to stabilize the pH and enhance the separation quality [32]. These innovations align with proprietary buffer kits used in impurity profiling by Camperi et al. [30], which streamline the detection of impurities with extended separation times of up to 100 min, emphasizing thorough impurity profiling for regulatory compliance.

**Figure 2:**
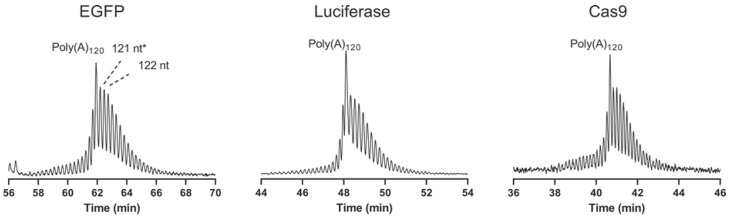
CGE-UV electropherograms of isolated poly(A) tails obtained from different mRNA constructs with poly(A)120 spikes. The most abundant peak is labeled with asterisk (*). Modified with permission from [29].

Recently, Di Grandi et al. also demonstrated the ability of CGE to achieve single-nucleotide resolution in the characterization of the 3′ poly(A) tail, down to 120 nucleotides (see Figure 2) [29]. Both poly(A) tail distribution and length can be determined by adding a synthetic poly(A) length marker to the sample. This approach can be easily qualified and used as a release method for the manufacture of mRNA-based therapeutics and vaccines.

However, despite its high potential for achieving mRNA size variant separation, CGE presents several limitations. The relatively long separation times, typically between 40 and 130 min, are a significant drawback, which may limit its practical application in routine quality control settings. Additionally, CGE requires careful optimization of experimental conditions such as capillary and buffer composition according to the type of quality attribute that is under investigation and as described above. To minimize these limitations, microchip capillary electrophoresis (mCE), a miniaturized platform of CE, has emerged as a robust technique for high-throughput mRNA analysis, offering rapid sample processing and minimal reagent consumption and requiring minimal method changes for optimization [28] (see Table 1). This method enables simultaneous analysis of multiple mRNA samples (~70 s per sample, and it can run a 96-well plate in approximately 2.5 h), making it ideal for large-scale production environments such as vaccine manufacturing. To determine whether this new high-throughput technology is sensitive and robust, a comprehensive comparison with conventional CGE instrumentation was carried out and demonstrated similar trends in mRNA purity, underlining the reliability and reproducibility of mCE results [30].

More recently, Rollo et al. explored the merits of microfluidic capillary zone electrophoresis (CZE) coupled to an MS detector for the characterization of short RNA molecules, including yeast tRNAPhe (75 nt) and HIV-1 5′-UTR (364 nt) from a digestion mixture prepared by treating the samples with RNase T1 [37]. It has highlighted the interest in using the development of microfluidic devices that combine both a capillary and a transmitter in the same chip, thus completely eliminating the need for an actual interface. Despite the novelty of this approach, improvements still need to be made, such as achieving CZE separation with a higher peak capacity, particularly critical for mRNAs producing a large number of fragments after digestion.

Finally, emerging research into circRNAs proposes expansions of CE’s capabilities. While not traditionally analyzed using CE, insight from recent electrophoretic studies on circRNA migration in gel (e.g., circular splicing products from linear precursor molecules, nicked circles, splicing intermediates, and excised introns) suggests the potential adaptation of CE for these novel RNA forms, broadening the scope of RNA therapeutics and diagnostics [38,39].

In conclusion, electrophoresis, particularly CGE, is indispensable for the comprehensive characterization of linear and circular mRNA. It ensures that mRNA therapeutics meet stringent quality control standards by evaluating mRNA integrity, size distribution, poly(A) tail length, and structural properties. However, the limitations of CGE, including long separation times, the need for optimization of conditions, and sensitivity issues, must be acknowledged, even if some progress is made to minimize the limitations. Moreover, mRNA synthesis frequently results in impurities, including truncated mRNA products and dsRNA, which can induce immunogenicity responses and reduce therapeutic safety and efficacy [30], and using CGE can be challenging in achieving high-resolution separation of short impurities or identifying the type of dsRNA, often necessitating the use of complementary technologies to ensure comprehensive analysis. As mRNA technology continues to evolve, electrophoresis will remain a key tool in optimizing the design, stability, and efficacy of RNA-based treatments.

## 3. Chromatographic Approaches

High-performance liquid chromatography is a versatile and essential analytical technique used in the characterization of mRNA [41]. Both LC software and hardware have been considerably improved over the past years. As of today, HPLC systems now operate at pressures ranging from 1000 to 1500 bar, utilizing ultrahigh-pressure liquid chromatography (UHPLC) to achieve either rapid or high-resolution separations. Manufacturers provide an extensive array of stationary phase morphologies, including sub-2 μm fully porous and superficially porous particles and nonporous materials. Additionally, they are actively developing specialized columns optimized for RNA analysis, leading to significantly better separation results compared with conventional LC columns. Anion exchange (AEX) chromatography, SEC, and IP-RP LC modes are the most often employed for mRNA analysis [41,42,43] and are often applicable to circRNAs. Recent developments are summarized in Table 2.

### 3.1. Anion-Exchange Chromatography

AEX is based on the reversible interaction between the negatively charged sugar-phosphate backbone of mRNA and the positively charged chromatography phase. AEX has been regularly used to purify mRNA [53] but also for the monitoring of IVT reactions to support mRNA manufacturing process development [44]. The monitoring of all IVT materials such as NTPs, Cap analogue, and plasmid DNA can be performed. Regarding the analysis of mRNA products, the highest-resolution AEX separation was performed using a pellicular particle stationary phase (PA200 column) and a neutral pH sodium chloride or sodium perchlorate salt gradient combined with separation at 60 °C or with a high-pH (pH = 12) salt gradient separation performed at 10 °C [45] (see Table 2). The use of denaturing conditions (high temperature/high pH) facilitates the separation by disrupting base-pairing and base-stacking interactions. Under these conditions, intact mRNA can therefore be linearized, enabling sharper chromatographic peaks to be separated and eluted.

**Figure 3:**
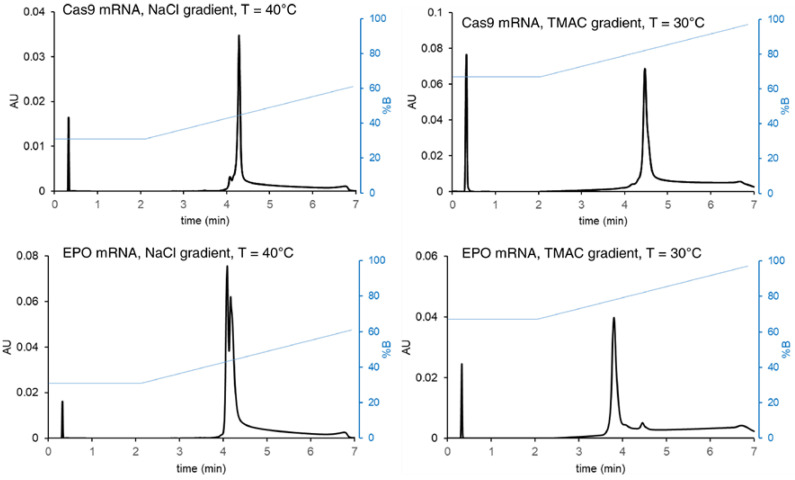
AEX separations obtained for the analysis of EPO (859 nts) and Cas9 mRNAs (4500 nts) using a classical NaCl gradient (**left panel**) versus those with an ion-pairing TMAC gradient (IPAX, **right panel**). Modified with permission from [46].

In parallel, the addition of an ion-pairing (IP) agent and relatively mild elution conditions have been suggested for alternative analytical methods [46]. An approach termed ion-pairing anion exchange (IPAX) utilizing a gradient of weak ion-pairing cations (e.g., tetramethylammonium chloride, TMAC) appears to provide different recovery and selectivity effects from conventional salt gradients [54]. TMAC can be employed as a mobile phase additive to form partially ion-paired mRNA molecules, and solutes can be eluted with less NaCl in the salt gradient. To date, IPAX has been performed with 25 mM HEPES and TRIS buffers (pH 7.5–8) and 1–3 M TMAC gradients. AEX separations using a classical NaCl gradient (left panel) versus those with an ion-pairing TMAC gradient are shown in Figure 3.

### 3.2. Size Exclusion Chromatography

The characterization of mRNA impurities remains a major analytical challenge. One area of concern is the potential impact of aggregates on the efficacy and safety of these vaccines. Therefore, the aggregate content should be measured to address any potential implications they may have on vaccine performance. To this end, SEC is one of the well-established HPLC methods for measuring aggregates of various types of molecules including antibodies [55], CRISPR-Cas9 gRNAs [56], and more recently mRNAs [30,48,57] (see Table 2). The advantages of using SEC include a simple sample preparation procedure, a quick turnaround time, and the possibility of being coupled with different detectors [56].

**Figure 4:**
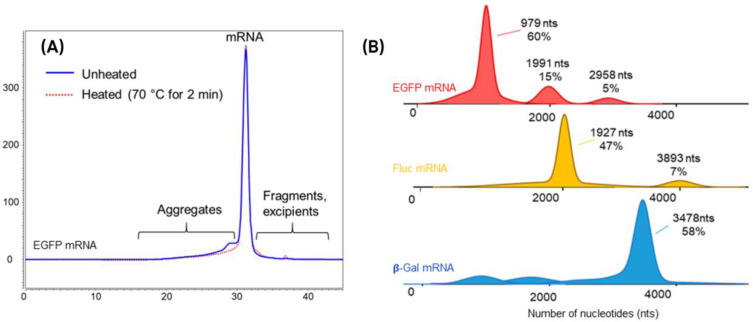
(**A**) SEC-UV profiles obtained for EGFP mRNA using an ultrawide pore column. The profiles were obtained before heating the samples (blue traces) and after heating (red traces). (**B**) Mass photometry profiles obtained for eGFP, Fluc, and β-Gal mRNAs. The number of nucleotides and the percentages of monomer, dimer, and trimer forms are indicated. Figures have been adapted with permission from [30,47].

With regard to mRNA analysis, it offers the possibility of characterizing it in its native form and can be used to determine its purity and explore aggregation states [58,59]. However, mRNA molecules are relatively large (e.g., 200–5000 nts) and therefore can easily be trapped in the pores of a typical SEC column, resulting in poor separation. As a result, many suppliers have recently introduced ultrawide pore SEC columns dedicated to the separation of mRNAs and lipid nanoparticles [47]. A typical SEC-UV profile, showing the separation of aggregates from the eGFP mRNA monomer, is shown in Figure 4A. The interest of using ultrawide pore SEC columns with average pore diameters of 1000 and 2000 Å for various lengths of mRNA samples was further investigated by D’Atri et al. [48]. This study demonstrated that an SEC column with 1000 Å pores proved to be optimal for the analysis of mRNA products, whatever the size between 500 and 5000 nucleotides. The addition of 10 mM magnesium chloride (MgCl_2_) in the mobile phase can be beneficial in improving the resolution and recovery of large size variants for some mRNA samples [48]. Because of SEC simplicity and the availability of ultrawide pore SEC columns with an improved resolution, it can become a useful tool for the analysis of mRNA aggregates [30,47,49]. Its application can be extended to an accurate determination of molecular weight (demonstrating an optimal separation of the species) and shape estimation when multi-angle light scattering (MALS) and online dynamic light scattering (DLS) are associated [47,49]. SEC-MALS can reveal important biophysical and structural information, which is often very difficult to obtain with other analytic methods due to the large size and high charge density of the mRNA molecules [51].

The nature of the aggregates (i.e., covalent or non-covalent aggregates) can be determined by heating the sample prior to analysis (see Figure 4A). Covalent and non-covalent aggregates are associated with 3′-loopback dsRNA sense-antisense dsRNA byproducts, respectively [30]. However, when it comes to distinguishing the various non-covalent forms (i.e., dimers, trimers, and more complex structures), SEC begins to run up against certain limitations. As shown in Figure 4A, the ultrawide pore SEC column does not allow separation of the different types of eGFP mRNA aggregates.

In this regard, mass photometry (MP) is a better alternative to characterize and differentiate these non-covalent forms. MP has emerged as a powerful tool to detect and measure single molecules in solution [60], offering intact mass measurements of mRNA and its complexes without labeling [30,49,51]. MP uses a contrast-enhanced interferometric scattering microscope for highly sensitive, precise, and non-invasive measurements. This technology is particularly useful for determining certain attributes of mRNA, such as molecular integrity, aggregates, and protein–mRNA interactions—key factors in therapeutic mRNA development. Similar to SEC chromatography, mass photometry can be used to measure mRNA purity, but it also offers the possibility to determine the average number of nucleotides [30,49,51]. A study has demonstrated MP’s repeatability and accuracy, distinguishing between mRNAs with subtle differences and showing robustness and wide mass range capability (i.e., 100–4000 nts) with low error rates [30]. Under stress conditions, MP detects dimer and trimer disruptions, confirming its utility as a stability-indicating method. Compared with SEC, MP does not require a large amount of mRNA sample (only 50 ng versus a few μgs) and does not suffer from poor separation resolution due to the limited pore size of SEC columns, enabling efficient separation of different aggregate forms. Examples of MP profiles obtained for different mRNA lengths (i.e., 900–3500 nts), showing the presence of monomers, dimers, and trimers, are presented in Figure 4B. Another study has confirmed that mRNA dimers measured in MP can be associated with sense–antisense dsRNA byproducts, contributing directly to immunological reactogenicity in different cell types [51]. Recently, Deslignière et al. demonstrated the interest of the MP approach to measure the mass of various intact high-mass capped mRNAs, up to 9400 nt (~3 MDa) in size, with high accuracy, while revealing low amounts of mRNA fragments and dimers [61]. Although MP is a relatively new technique, it has great potential to become a valuable, rapid, and simple orthogonal method for characterizing mRNA therapeutics in real time. With these promising results, further research is needed to fully establish MP technology in the mRNA field.

### 3.3. Slalom Chromatography

**Figure 5:**
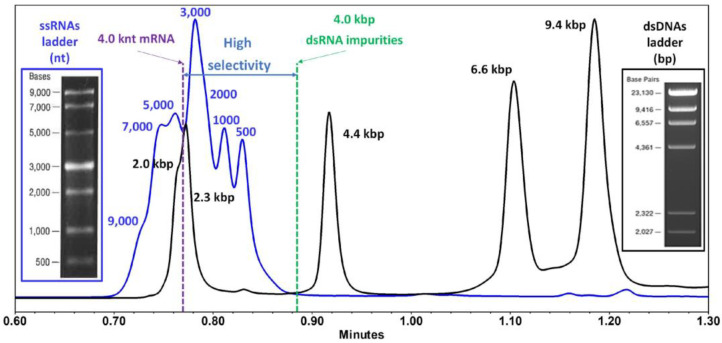
Proof of concept for the application of the SC retention mode for the baseline separation of the main 4.0 knt mRNA product (see blue ssRNA ladder) and 4.0 kbp dsRNA impurities (see black dsDNA ladder), one important byproduct of IVT processes to be quantified. Adapted with permission from [63].

Slalom chromatography (SC), initially co-discovered by Boyes and Kasai in the late 1980s, has recently re-emerged as a breakthrough technique to rapidly analyze large DNA and RNA samples. It is offered as a promising alternative to the MP method for the separation of sense–antisense dsRNA byproducts. SC separates molecules based on hydrodynamic forces rather than traditional equilibrium-based interactions [62]. Recent studies have reviewed key equations that explain how DNA/dsRNA chains stretch under force, relax back into their natural coiled state, and move through the chromatography column. They also explore how retention time is influenced in hydrodynamic chromatography (HDC-SC) and the factors affecting separation efficiency, such as theoretical plate height [63]. Gritti recently demonstrated a proof-of-concept study showcasing SC as a rapid method (under 1 min) for separating immunogenic dsRNA impurities generated during the IVT process used in mRNA therapeutics [63]. The study also assessed dsRNA structural heterogeneity, including conformational isomers. As shown in Figure 5, sense–antisense dsRNA species exhibit significantly higher retention than IVT mRNA. This occurs because mRNA is excluded via an HDC mechanism, while dsRNA is retained through an SC mechanism. However, further advancements, such as the development of mesoporous particles, are needed to commercialize SC columns for broader applications [63].

### 3.4. Ion-Pair Reversed Phase Liquid Chromatography

IP-RP LC is commonly used for purity determination of mRNA [43]. It is particularly effective for separating abortive transcripts and mRNAs with partial poly(A) tails from the full-length mRNA product [30]. IP-RP LC typically involves high column temperatures (i.e., 70–80 °C) and the use of ion-pairing agents in association with organic solvents, in particular methanol [64]. The positively charged IP agent complexes with the RNA negatively charged backbone, enabling an increased affinity of the RNA molecules on the RP stationary phase. The choice of IP agents has been fine-tuned through extensive research. As listed in Table 2, triethylammonium acetate (TEAA), hexafluoroisopropanol (HFIP), and N,N-diisopropylethylamine (DIPEA) are the most employed [30,43,45,50,65,66]. Regarding the choice of RP columns, most of the studies employed C18 phases but also newly stationary phases, such as divinylbenzene-based columns and supermacroporous polymer resin, offering enhanced performance for mRNA separations [42]. Generally speaking, mRNA molecules require low-binding materials such as PEEK or any other sort of bio inert columns due to their trend to absorption. In addition, particular attention to the column pore size is critical for suitable analysis since mRNA can reach up to 5000–10,000 nucleotides, which can prevent proper analysis with traditional columns, not matching RNA hydrodynamic radii.

Several studies reported the stability indicating power of IP-RP LC for mRNA and circRNAs, upon different types of stress conditions, including exposure to heat, hydrolytic conditions, and treatment with ribonucleases [50,52]. Stability studies are typically required for RNA-based drugs by regulatory agencies. Currie et al. showed that the formed degradation products appeared as shorter RNA fragments in front of the main peak and could be well monitored, and the relative stability of the mRNA under the various stressed conditions could be assessed [50]. Moreover, Cheng et al. also reported an IP-RP LC method aiming at the identification of target circRNAs, product-related substances, and impurities [52]. Degradation patterns of circRNAs under thermal acceleration conditions and biological analysis of degradation products were investigated. Recently, a study demonstrated that both IP-RP LC and CGE techniques are effective in assessing the purity of mRNA species, revealing strong similarities in their separation patterns [30]. However, while both methods are capable of detecting the presence of an mRNA variant without a poly(A) tail and discriminating species containing a partial poly(A) tail in the pre-peak region, the distinction becomes clear for impurities present in the post-peak region, where CGE exhibits higher resolution due to its stronger denaturation conditions than IP-RP LC [30]. This study also highlights the importance of using orthogonal methods to monitor a single quality attribute such as purity, offering a greater guarantee of detecting minor impurities [30].

**Figure 6:**
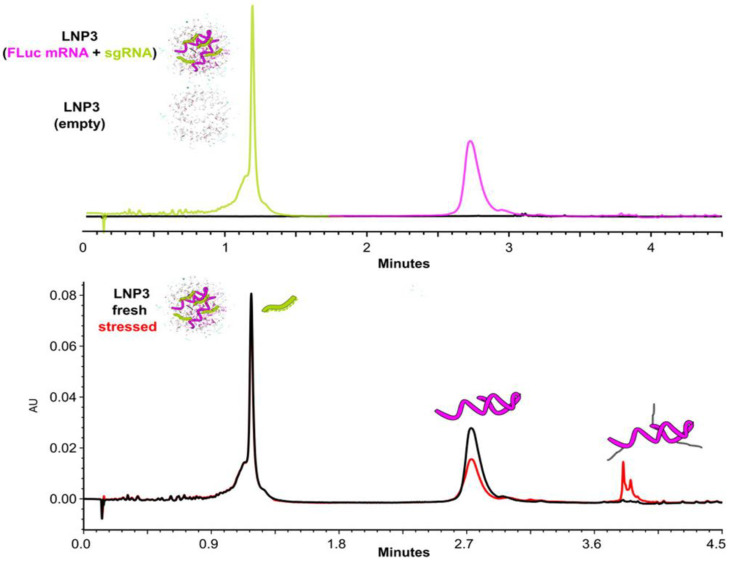
IP-RP chromatograms showing the response of LNP loaded with FLuc mRNA and sgRNA or formulated without any payload (empty—black trace). Overlay of IP-RP chromatograms for a fresh LNP sample (black) and a LNP sample stored at room temperature for 1 month (red) showing separation of lipid-adducted mRNA and loss of intact mRNA peak. Modified with permission from [68].

Moreover, IP-RP LC can be used to analyze and quantify mRNA in an LNP formulation, enabling the integrity and purity of the mRNA cargo to be assessed by separating it from other LNP components based on its charge and hydrophobicity, thus providing valuable information for QC in the development of mRNA-based therapies [67,68]. To this end, a complete extraction of RNA from the carrier is required for quantification of the RNA cargo of nanoparticles. A number of solvents have been employed for extracting RNA from nanoparticles, e.g., non-ionic detergents, organic solvent/aqueous phase mixtures, and phenol:chloroform:isoamyl alcohol [69,70]. Non-ionic detergents are the most widely used extraction reagents due to the effective extraction of nucleic acid cargos from lipid-based nanocarriers. However, detergents are detrimental to HPLC columns, and the LC flow path and can lead to signal suppression. Cargos based on sgRNA [66] and mRNA [47] have been quantified using IP-RP LC, but the simultaneous quantification of multiple co-loaded RNA cargos in delivery systems remains a challenge [69]. Recently, an innovative detergent-based deformation process and IP-RP separation have been developed to characterize nucleic acid payloads, ranging from the small interfering RNA to Cas9 mRNA [67,68]. To enable direct measurement of double nucleic acid payloads, the use of a deforming SEC mobile phase composed of low levels of sodium dodecyl sulfate and isopropanol is used. This enables intact LNP samples to be injected directly into the LC flow path, where they are dissolved in their individual components [68]. As shown in Figure 6, the IP-RP LC method has sufficient resolution to separate impurities from mRNA adducts and provide information on mRNA quality in addition to quantification, within a 12 min analysis.

The main benefit of IP-RP LC over other chromatographic modes (such as AEX, SEC, and SC) is that it can be easily coupled with MS, considerably broadening its field of application. IP-RP LC-MS is often used for measuring important structural features of mRNA molecules, such as 5′ capping efficiency and 3′ poly(A) tail length [43]. Structural characterizations for the capping efficiency and the poly(A) tail by LC-MS have been recently reviewed [41,43]. Briefly, 5′ capping analysis involves several sample preparation steps that include the generation of predefined fragments from the 5′ end of mRNA molecules using RNase H, DNAzyme, or ribozyme and the enrichment of the 5′ cleavage products, followed by the IP-RP LC-MS analysis [71,72]. For poly(A) tail analysis, poly(A) tails are cleaved from IVT mRNA using the enzyme RNAse T1 and analyzed by IP-RP LC-MS, which provides information on the tail length at a single-nucleotide resolution [10,11,71]. In some cases, it may be desirable to purify the poly(A) tail mixture prior to LC-MS analysis. To this end, after digestion with RNase T1, poly(A) tails can be purified by hybridizing them to oligo(dT)25-coated magnetic beads prior LC-MS analysis [51,71]. However, the need for multiple sample preparations and analysis methods for each mRNA attribute is not optimal.

To overcome this hurdle, Wang et al. demonstrated the value of using DNAzymes adapted for the selective cleavage of mRNA at the G-U dinucleotides in the UTRs, releasing both the 5′-capped or uncapped short fragment and the 3′-Poly(A) tail cleavage product [72]. The mRNA fragments cleaved from the digestion reaction by the 5′- and 3′-DNAzymes were then analyzed directly using IP-RP LC for simultaneous characterization of the cap and poly(A) tail, without additional purification steps and costly MS analysis, clearly streamlining the sample preparation and analysis process, making it highly suitable for QC testing.

Last, it should be noted that enzymatic digestion of mRNAs followed by an LC-MS/MS analysis for oligonucleotide sequencing have attracted growing interest and will be discussed in the next section.

## 4. Mass Spectrometric Approaches

Mass spectrometry has become an invaluable tool for the characterization of biopharmaceuticals, particularly for antibodies and novel protein-based formats due to advancements in instrumentation and bioinformatic tools [73]. Advantages and limitations of mRNA characterization by MS have been recently reviewed [41,43,74]. In this section, a general overview of the principles for mass determination by MS is provided, and the potential of MS for the analysis of mRNA quality attributes is highlighted for selected recent studies (see Table 3).

MS-based characterization of mRNA is an emerging field compared with the advancements in the analysis of therapeutic proteins that have been achieved within the last decades. Mass spectrometers are detectors that allow determining the mass of molecules by measuring the mass to charge ratio (*m*/*z*) of molecules upon ionization in a negative mode (negatively charged ions) or a positive mode (positively charged ions). MS instruments differ in the ion source, *m*/*z* analyzer, and detector. The mass determination of an analyte by MS requires the charge (*z*) information that may be derived from the spacing between isotopes, if isotopic resolution is achieved, or charge state distribution, if resolved for average masses. More recently, charge detection MS (CD-MS) has been explored and allows the determination of the mass of very heterogeneous and large analytes, where conventional charge determination is not possible [75,76,77]. In addition, MS instruments are commonly equipped with capabilities to specifically fragment selected ions of molecules via various techniques (MS/MS or MSn), which can be applied for bottom-up or top-down sequencing.

Electrospray ionization (ESI) is a soft ionization technique used in MS, where the analyte is introduced either through a continuous flow via a syringe pump (direct infusion) or directly via an emitter needle (static spray). Additionally, ESI-MS can be seamlessly integrated with electrophoretic or chromatographic separation techniques for enhanced analytical performance [78]. MS usually requires the use of “MS-friendly” volatile salts and solvents to allow for efficient ESI and sufficient MS spectra quality. The experimental conditions for ESI can be separated into “denaturing” conditions, which usually apply low pH and/or organic solvents, disrupting inter- and intra-molecular interactions, and conditions that maintain structural elements, such as aggregates, called “non-denaturing” or “native” conditions, which usually use neutral pH and aqueous solvents [73,78]. MS offers high sensitivity, mass accuracy, and mass resolution that make it a powerful technology for the characterization of complex molecules with a high degree of heterogeneity, such as mRNA. In contrast to MALS/DLS or MP analyses, which were discussed previously, co-eluting or co-existing variants with much smaller mass differences can be resolved allowing the achievement of higher mass accuracies. Hence, MS allows for more confident mass assignments, which provides great potential to improve the analysis of mRNA impurities and integrity.

**Figure 7:**
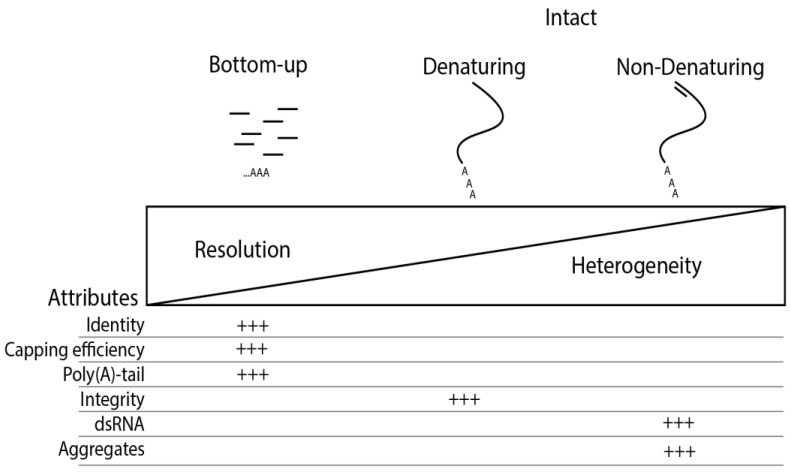
Schematic representation of different approaches for the MS analysis of mRNA variants and the attributes that can be resolved.

The potential heterogeneity of mRNA is significantly higher compared with proteins and comprises several contributing factors such as poly(A) tail heterogeneity and mRNA impurities including abortive transcripts, nucleotide modifications, or dsRNA. To better understand the mRNA heterogeneity of the full-length mRNA at an intact level, information from other MS-based approaches using nucleases is required to analyze smaller fragments or larger fragments (i.e., Poly(A) tail) via a bottom-up approach (Figure 7). While the reduced complexity in bottom-up approaches provides higher resolution and is required for confident and accurate determination of specific quality attributes, such as capping efficiency or nucleotide modifications, other quality attributes of full-length mRNA can only be achieved at the intact level. While the separation of bottom-up approaches hyphenated to MS has been demonstrated in recent years, the MS hyphenation of separation techniques at the intact mRNA level remains an analytical challenge that has a high potential to drastically advance the understanding and characterization of mRNA impurities.

### 4.1. Advancements in Oligonucleotide Mapping

**Figure 8:**
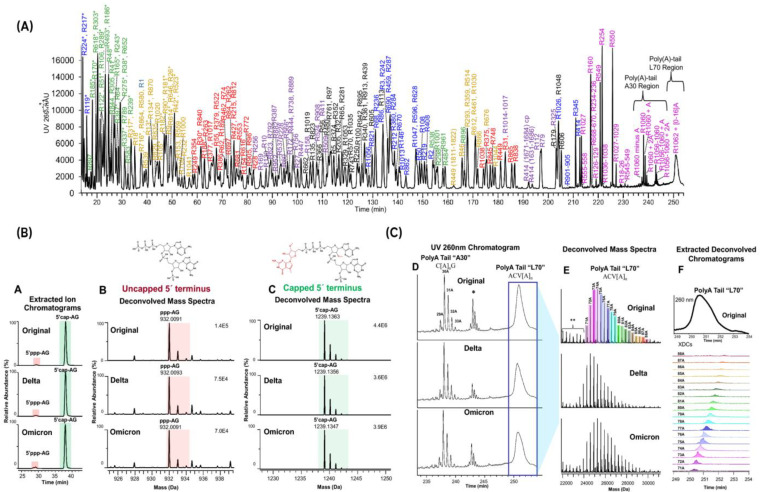
Oligonucleotide mapping via MS to enable comprehensive primary structure characterization of an mRNA vaccine against SARS-CoV-2. (**A**) IP-RP LC-MS RNase T1 oligonucleotide map of BNT162b2 mRNA. “*” denotes a sequence-repeat oligonucleotide, where the single peak assignment represents all identical oligonucleotides in the sequence. Each color distinguishes the number of nucleotides per digestion product: blue, 4, 10, and 16; green, 5 and 11; gold, 6 and 12; red, 7 and 13; purple, 8 and 14; black, 9 and 15: magenta, >16. (**B**) (1) Extracted ion chromatograms of uncapped (5′ppp-AG) and capped (5′ cap-AG) versions of the 5′ terminal oligonucleotide for BNT162b2 variant constructs Original, Delta, and Omicron. (2, 3) Deconvoluted mass spectra of uncapped (5′ppp-AG) and capped (5′ cap-AG) versions, respectively, of the 5′ terminal oligonucleotide for BNT162b2 variant constructs Original, Delta, and Omicron. (**C**) UV chromatograms of the poly(A)tail region for BNT162b2 variant constructs Original, Delta, and Omicron. The blue box highlights the chromatographic distribution of L70 poly(A) oligonucleotides (unseparated), which are further described in panels (5) and (6). (5) Deconvoluted mass spectra of the L70 poly(A) oligonucleotide distribution. (6) Extracted deconvolved chromatograms of BNT162b2 Original L70 poly(A) oligonucleotide distribution highlighted in panel (5). Modified with permission from [79].

For oligonucleotide mapping or bottom-up workflows, mRNA digestion is commonly performed by RNases (mainly RNase T1), followed by an LC-MS/MS analysis using negative mode. Enzymatic digestion using ribonucleases such as RNase T1, which cleave after guanosine residues in unpaired regions of the RNA, and RNase A, which cleave after pyrimidine ribonucleotides, generates smaller oligoribonucleotides that are more amenable to chromatographic separation and MS analysis. Gao et al. demonstrated the value of such a workflow to support the development of Comirnaty, the world’s first commercial mRNA vaccine that immunizes against the SARS-CoV-2 virus [79]. This approach ensures the quality, safety, and efficacy of mRNA vaccines by providing confirmation of the construct identity and primary structure. Moreover, this approach enables the simultaneous characterization of the 5′ cap and poly(A) 3′ length, which are important quality attributes for a vaccine or mRNA-based therapeutic [79] (Figure 8). Interestingly, the authors reported the interest to optimize MS/MS fragmentation conditions for proper identification of RNA fragments. For the BNT162b2 mRNA, the authors noted that of the 302 unique nucleotides sequences generated by an in silico RNase T1 digestion, 220 of the unique nucleotide sequences are isomers. These species share the same composition, and therefore mass, with at least one other oligonucleotide sequence, and many differ only by a single nucleotide exchange between two positions. Therefore, high-quality MS/MS spectra and accurate spectral assignments are required to avoid false-positive identifications. In this study, the authors optimized the higher-energy collisional dissociation (HCD) conditions to find a balance of relatively abundant terminal fragment ions for sequencing and mitigation of internal fragmentation, ensuring that the most sequencing-informative MS/MS spectra are acquired (see Table 3).

To overcome the barriers associated with producing large numbers of small RNA fragments, which correspond to many different locations in the mRNA sequence and therefore do not generate unique sequences, other efforts have focused on improving sample preparation [80,81,82,83,84]. For example, Jiang et al. demonstrated a combination of several digestion enzymes with alternate cleavage behaviors, such as olicin E5 and maz5, in addition to RNase T1 in order to have complementary sequence coverages [80]. The cleavage sites for colicin E5 and mazF enzymes are GU and ACA sites, respectively. Using this approach, greater than 70% sequence coverage was achieved on mRNAs near 3000 nucleotides long. For instance, the sequence coverage for Epo mRNA (859 nts) increased from 52.9% using RNase T1 alone to 86.6% when all digestions were combined. However, unlike RNase T1, the authors reported that both colicin E5 and mazF exhibited condition-dependent minor digestion products and showed that the salt concentration and pH affected the abundance of minor digestion products, which can have an impact on assay repeatability. Similar parallel nuclease digests using alternative RNases have also recently been employed to sequence mRNA using an online LC-MS platform [83]. Once again, the study showed that combining digestion enzymes increases confidence in RNA fragment assignment. Sequence coverages of using unique digestion products were between 5.8–51.5 and 3.5–19.3% for RNase T1 and RNase A, respectively. With the nonunique digestion products, the sequence coverage was increased to 65.6–85.6 and 69.7–85.0% using RNase T1 and A, respectively. As pointed out, current limitations of oligonucleotide strategy still reside in terms of ambiguities between the small RNA fragments.

Another strategy is to perform controlled partial digestion to induce missed cleavages in order to create longer fragments that could be uniquely matched to the original RNA sequence [81,84]. Partial RNase digestions can be performed in solution or using RNase T1 immobilized on magnetic particles [81] prior to high-resolution LC–MS/MS. Using oligonucleotide data analysis software, the automatic identification of multiple missed cleavages in conjunction with accurate mass analysis and MS/MS fragmentation spectra enabled the acquisition of high sequence coverage based on the corresponding RNA sequence. Using this approach, Vanhinsbergh et al. achieved a sequence coverage higher than 80% of various mRNA sequences (up to 2000 nts for Fluc mRNA), which was generated from a single partial RNase T1 digest [81]. More recently, Tang et al. reported the use of a flow through (FT)-based digestion strategy, relying on immobilized RNases, which significantly boosted the overall sequence coverage (over 93%) of therapeutic mRNAs of varying sizes (900–4500 nts). The use of an automated digestion workflow using an AssayMAP platform offered significant advantages in method reproducibility and throughput [84].

In another study, Nakayama et al., 2022 reported a novel analytical platform based on an isotope-dilution nanoflow LC–MS method that quantitatively characterizes long, modified mRNAs (200–4300 nt) by comparing them with a stable isotope-labeled reference with an identical sequence to that of the target RNA drug [82]. This platform includes database searching using the mass spectra as a query, enabling the confirmation of the primary structures of different mRNAs, with sequence coverage at 100%. This approach allows the detection/identification of defects in the sequences and the measurement of the efficiencies of the 5′ capping and integrity of the poly(A) tail. While this approach offers great potential, it also has its limitations. As the authors explain, the method is not suitable for quantifying capping efficiency when a post-transcriptional capping approach is used. Furthermore, when analyzing longer RNAs, the approach may suffer from not being able to confirm the identification of all RNA fragments. Nevertheless, it allows the demonstration of identity to the standard, offering the possibility of confirming the sequence identity of mRNA drugs at different times, i.e., for batch verification [82].

In addition to the IP-RP LC mode, hydrophilic interaction liquid chromatography (HILIC) separations have also attracted growing interest for the separation of short oligonucleotides and have shown promise for the mapping of mRNA sequences in combination with MS [85]. HILIC separation offers three main benefits over IP-RP separations, notably, a significant reduction in mobile phase cost, improved mobile phase stability for MS applications, and the absence of ion-pairing agents to ensure satisfactory MS detection performance. Goyon et al. recently reported nucleotide mapping of mRNAs using an online 2D LC-MS system, where RNase reactors (RNases T1 and A) are placed online with a HILIC column [83]. Various mRNAs of varying lengths and chemical modifications were characterized. Sequence coverages of 5.8 to 51.5% and 3.7 to 50.4% were achieved for the online and offline approaches, respectively. Interestingly, Hayashi et al. introduced an ammonium bicarbonate-based non-IP-RP LC-MS method for analyzing oligonucleotide therapeutics (OTs), demonstrating comparable sensitivity and quantification ranges to existing IP-RP LC-MS methods while potentially offering advantages like reduced reagent issues and cost [86]. As far as HILIC profiles are concerned, fewer peaks are generally observed compared with IP-RP LC profiles for a given mRNA sequence, which may suggest a lower peak capacity for oligonucleotide separation. Further studies should be carried out to fully adapt the use of the HILIC mode to the characterization of larger mRNAs.

Last, Mutchek et al. recently demonstrated the advantages of mid-level sequencing of mRNA using strand-cleaving deoxyribozymes with no front-end separation [87]. Deoxyribozymes, in the presence of appropriate co-factors in aqueous solution, were found to show an excellent selectivity for the hydrolysis of mRNA. The main advantage in mid-level approaches is the simplification by reducing the number and ambiguity of cleavage products. For example, cleavage of the 758 nt eGFP mRNA resulted in only 10 unique products that facilitate unambiguous identification. In contrast, a more common RNase T1 digestion would result in 87 unique cleavage products. In conclusion, the design of new cleavage enzymes in combination with advancements in MS analysis and potential for combining evolving separation technologies is expected to improve the possibilities to study mRNA quality attributes.

**Table 3:** Recent MS-based applications for mRNA characterization.

RNA/ Lenght (nt)	Analysis Level	Analytical Method Conditions				Target QA	General Comments	Ref.
		LC Mode	LC Column	Mobile Phase	MS/Software for Data Analysis			
SARS-CoV-2 mRNA vaccine	Oligo mapping	IP-RP	ACQUITY Premier Oligonucleotide C18 (130 Å, 1.7 µm, 2.1 × 150 mm)	MPA: 0.1% TEA, 1% HFIP, H_2_O MPB: 0.1% TEA, 1% HFIP, MeOH	Orbitrap Exploris 240 MS/Biopharma Finder	Sequence confirmation, mRNA chemistry modifications, 5′-capping efficiency, and 3′ poly(A)-tail length	100% maximum sequence coverage, optimized MS/MS HCD fragmentation to sequence isomers.	[79]
- Epo (859 nts) - FLuc (~2000 nts) - α-catenin (900 nts)	Oligo mapping	IP-RP	ACQUITY UPLC Oligonucleotide BEH C18 (130 Å, 1.7 μm, 2.1 × 100 mm)	MPA: is 1% HFIP, 0.1% DIPEA, H_2_O MPB: 0.075% HFIP, 0.0375% DIPEA, 65% ACN, 35% H_2_O	6550 Q-TOF MS/Agilent MassHunter data	Sequence confirmation and sequence impurities (SNPs)	Use of multiple endonucleases (RNase T1, colicin E5, and mazF), enabling complementary sequence coverage.	[80]
eGFP, SARS-CoV-2 spike protein mRNA, Fluc (5moU) mRNA	Oligo mapping	IP-RP	DNAPac RP (150 mm × 2.1 mm)	MPA: 0.2% TEA and 50 mM HFIP MPB: 0.2% TEA, 50 mM HFIP, and 20% *v/v* ACN	Orbitrap Exploris 240 MS/Biopharma Finder	Sequence confirmation and impurity analysis	Partial RNase digestions using RNase T1 immobilized on magnetic particles >80% sequence of coverage.	[81]
p- and s-mimBNT162b2 mRNAs	Oligo mapping	IP-RP	RP Develosil C30-UG column (3 μm particle size, 150 μm × 240 mm)	MPA: 10 mM TEAA, pH 7, in (90:10, *v/v*) H20, MeOH MPB: (60:40, *v/v*) 10 mM TEAA, ACN	Q Exactive Orbitrap MS	5′ capping efficiency and 3′ poly(A)-tail length	Isotope-dilution LC–MS method to sequence 200–4300 nts mRNAs Direct Nanoflow LC-MS/MS.	[82]
eGFP, eGFP (5moU), Fluc, Nickase Cas9 (5moU), and Cas9	Oligo mapping	HILIC	1D: immobilized RNase T1, and RNase A cartridges (2.1 × 33 mm) 2D: Premier BEH amide column (130 Å, 1.7 μm, 2.1 × 50 mm)	MPA: 10 mM NH4OAc in H_2_O/acetonitrile (3:97, *v/v*) MPB: 25 mM NH4OAc in H_2_O/ACN (60:40, *v/v*)	Q-Exactive Orbitrap MS/Biopharma Finder version 5.0 software	Sequence confirmation, mRNA chemistry modifications	Online nucleotide mapping of mRNAs using 2D LC-MS system with an 1D immobilized RNase cartridge, followed by HILIC-MS analysis.	[83]
eGFP, eGFP (5moU), Epo (5moU), and Cas9 (5moU)	Oligo mapping	IP-RP	Oligonucleotide BEH C18 (130 Å, 1.7 μm, 2.1 mm × 150 mm)	MPA: 1% HFIP, 0.1% DIPEA in H_2_O MPB: 0.075% HFIP, 0.0375% DIPEA in 65% ACN, 35% H_2_O	Thermo Q-Exactive plus MS/Byonic software—Byologic “Digested Oligonucleotides”	Sequence confirmation, mRNA chemistry modifications	Flow through-based strategy to achieve the limited RNase T1 digestion, which boosted the overall sequence coverage (over 93%). Automated digestion workflow using the AssayMAP platform.	[84]
- EPO (859 nts) - Fluc (~2000 nts)	Poly(A) tail analysis	IP-RP	ACQUITY Premier Oligonucleotide C18 (130 Å, 1.7 μm, 50 × 2.1 mm)	MPA: 8 mM DIPEA, 40 mM HFIP in H_2_O, pH 8.8 MPB: 4 mM DIPEA, 4 mM HFIP in 75% EtOH	Waters BioAccord LC MS system, MassLynx 4.2	Poly(A) tail length and heterogeneity	Evaluation of IP RP LC method for poly(A) tail length measurement, demonstrating robustness and suitability for routine mRNA quality control.	[10]
- Largest mRNA: eGFP (758 nts)	Mid-level oligonucleotide	No separation, nano ESI	In-house pulled quartz emitters	150 mM NH4OAc	Bruker solariX XR FTICR MS equipped with a 7 T magnet, Bruker DataAnalysis, RiboDynamics, SeqRead	Mid-level mRNA sequencing with CID	Proof-of-concept study for mid-level mRNA sequencing using RNA-cleaving deoxyribozymes. Decreased complexity for sequencing larger fragments and increased assignment confidence.	[87]
- Poly(A) tail: T1 cleavage, 100 nts in-house produced -mRNA +/- Poly(A) tail (783/683 nts) - IP-RP fractionated mRNA (580 nts)	Intact mass analysis, Poly(A) tail analysis	No separation, static ESI	Borosilicate emitter	Buffer: 200 mM NH4OAc	Orbitrap Q Exactive UHMR MS, FreeStyle (v 1.8) for isotopically resolved Poly(A) tail, UniDec (v 6.0.3.) for deconvolution of intact mRNA data	Integrity, PolyA heterogeneity	Isotopic resolution of Poly(A) tails up to 100 nts under native MS conditions in positive mode. Intact mass analysis mRNA, revealed new insights on integrity from IP RP LC fractions.	[30]
- mRNAs with/without Poly(A) tail using 5 different T7 polymerases	Intact mass analysis, Poly(A) tail analysis	No separation, static ESI	Borosilicate emitter	Buffer: 200 mM NH4OAc + 100 mM TEAA for charge reduction	Orbitrap Q Exactive UHMR MS, UniDec (v 6.0.3.) for deconvolution of data	Integrity, poly(A) tail, 5′loopback ds dsRNA	Functionality testing correlation Native MS analysis can detect short 3′loopback dsRNA impurities.	[51]

ACN, acetonitrile; BEH, bridged ethyl hybrid; DIPEA, N,N-diisopropylethylamine; Epo, human erythropoietin; EtOH, ethanol; Fluc, firefly luciferase; FTICR, Fourier-transform ion cyclotron resonance; HFIP, 1,1,1,3,3,3-hexafluoro-2-propanol; HILIC, hydrophilic interaction liquid chromatography; IP-RP LC, ion-pairing reversed phase liquid chromatography; MeOH, methanol; MPA, mobile phase A; MPB, mobile phase B; MS, mass spectrometry; N/A, not applicable; NH4OAc, ammonium acetate; Q-TOF, quadrupole time-of-flight mass spectrometer; SNPs, single-nucleotide polymorphisms; TEA, triethylamine; UHMR MS, ultrahigh mass range mass spectrometry; UV, ultraviolet; 5moU, 5-methoxyuridine.

### 4.2. Advancements in Intact MS-Based Approaches

While MS-based approaches have been widely applied to characterize mRNA structural features, such as poly(A) tail length, distribution [10,11,71], and capping efficiency [9], using oligonucleotide mapping applications (see Table 3), a limited amount of studies have been reported using MS for the analysis of intact mRNAs (i.e., >200 nts) [57,88]. Brophy et al. reported the characterization of EPO mRNA (858 nts), Fluc mRNA (1909 nts), and Cas9 mRNA (4521 nts) using IP-RP LC-TOF MS and CD-MS methods [57]. However, the authors reported still facing challenges for the analysis of large mRNAs due to the limited resolution of congested charged states and associated spectra complexity. Generally, the adduct formation propensities of mRNA and inherent mRNA heterogeneity are the major challenges for intact analysis [89]. For MS analysis, denaturing conditions and negative ionization ESI-MS mode are commonly applied due to mobile phase compositions in IP-RP LC-MS approaches. Positive ionization of short intact oligonucleotides has recently been demonstrated (25 nts, intact mass < 10 kDa) but revealed poor spectral quality even for short sequences [89].

**Figure 9:**
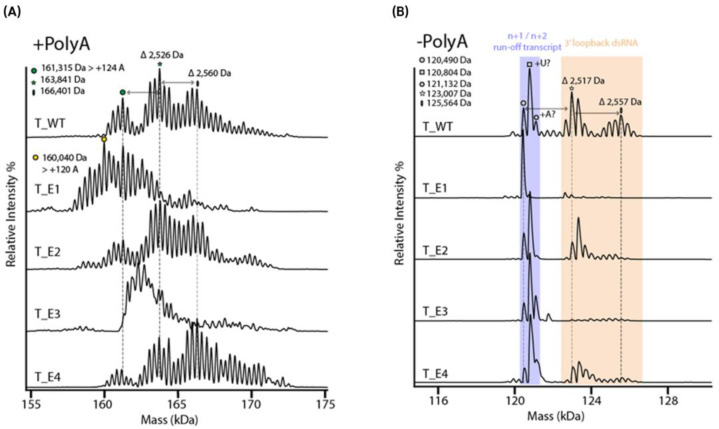
Representative intact mass analysis of mRNA variants by native MS. Superposition of the deconvoluted spectra of mRNA (**A**) with and (**B**) without poly(A) synthesized by five different T7 polymerases. WT, wild-type T7; E1–E4, four engineered T7 polymerases. n + 1/n + 2 transcripts are represented in blue, and the presence of 3′-loopback dsRNA is represented in orange. Adapted with permission from [51].

However, the analysis of intact mRNA by MS may provide unique insights into its integrity due to the high specificity that is provided. The major heterogeneity of the mRNA mass profiles is mostly related to the poly(A) tail. Recently, native MS in positive mode was applied to compare mRNA produced with/without poly(A) tail and confirmed that the complexity is drastically reduced for mRNA without a poly(A) tail [30] (Figure 9). Native MS can also be useful to resolve other attributes, such as n + 1/n + 2 transcripts or dsRNA impurities. Due to the high resolving power of MS and the applied non-denaturing conditions, variants with mass increments of +2.5 kDa and +5 kDa, corresponding to the addition of 8 and 16 nucleotides, were assigned as 3′-loopback dsRNA and were found to be dependent on the applied T7 polymerase that was used for mRNA generation [51]. This substantiates the high potential of high-resolution native MS for applications of mRNA characterization [90]. Other techniques, such as ELISA, that are used to determine dsRNA are limited to detecting up to 40 nts impurities, while MS has the potential to provide up to single-nucleotide resolution. In addition, the authors used charge reduction via triethyl ammonium acetate to increase the resolution between charge states, which facilitated the comparison of intact mRNA comprising poly(A) tails. However, the studies did not use upfront separation in direct hyphenation to MS, which is expected to resolve many more mRNA variants and increase the assignment confidence due to the added separation dimension.

Recently, microchip CE-MS was applied to analyze shorter oligonucleotides (up to 25 nts) in positive ionization mode under native MS conditions [88]. This approach remains to be explored for applications of larger mRNA. In addition, recent efforts in establishing CD-MS for analyzing heterogeneous analytes show high potential to be applied to reveal the complexity of large mRNAs at an intact level [61]. In a recent study, the authors demonstrated how single-particle Orbitrap-based native CD-MS can measure the mass of various intact mRNAs. However, they reported that while ensemble MS gave approximate masses for mRNAs < 2000 nt, it did not provide information on mRNAs with longer sequences. To circumvent the drawbacks of Orbitrap-based native CD-MS, the authors showed the interest to record individual ions. Low-charge mRNA components exhibited unstable ionic behavior, hampering initial CD-MS measurements, while high-charge populations offered a better signal-to-noise ratio and reduced charge uncertainty, with radically improved mass accuracy [61]. Finally, the combination of separation techniques, such as SEC and CD-MS as reported previously for the analysis of large proteins and virus-like particles, has great potential to provide novel insights into mRNA quality attributes, for example, the composition of mRNA aggregates [75].

## 5. Advanced mRNA Sequencing Approaches

Sanger sequencing has long been considered the gold standard for analyzing mRNA. However, in recent years, next-generation sequencing (NGS) technologies have revolutionized our understanding of mRNA biology, offering unprecedented insights into its molecular intricacies. The advent of short-read and long-read sequencing platforms, such as Illumina, PacBio, and Oxford Nanopore Technologies (ONT), has empowered researchers to investigate critical mRNA quality attributes, including sequence identity, integrity, purity, poly(A) tail length, and nucleoside modifications. Each platform brings unique strengths: Illumina excels in precision and throughput, PacBio offers high accuracy for full-length transcripts, and ONT provides the versatility of direct RNA sequencing, enabling real-time analysis without amplification. By leveraging these complementary capabilities, scientists can optimize mRNA vaccine formulations to enhance their efficacy and safety. This section explores the specific contributions and future potential of these sequencing technologies in advancing mRNA vaccine research, focusing on the innovative methodologies shaping the future of vaccine development. Table 4 summarizes the different sequencing methods available for assessing mRNA quality.

### 5.1. Sanger Sequencing

Traditionally regarded as the gold standard for mRNA sequence verification, Sanger sequencing was established over three decades ago and offers robust GMP solutions. However, it has notable limitations. This method involves converting mRNA into cDNA via reverse transcription, amplifying the cDNA through PCR, and sequencing the amplified DNA using primers. Sanger sequencing is labor intensive and less effective for analyzing large or complex sequences. Designing primers for specific regions can be challenging, particularly for full-length transcripts, and the method has limited sensitivity for detecting low-frequency variants or contaminants. In contrast, NGS technologies overcome many of these limitations by offering higher throughput, greater sensitivity, and the ability to analyze full-length sequences without the need for amplification or extensive primer design. While Sanger sequencing remains useful for targeted verifications, the advancements offered by NGS have made it a more robust and scalable option for comprehensive mRNA quality assessments.

### 5.2. Illumina Sequencing for Short-Read Precision

Illumina mRNA sequencing is a widely used NGS technology for transcriptomic studies. This method typically begins with isolating mRNA from a sample, which is then reverse-transcribed into complementary DNA (cDNA) to create sequencing libraries. Using Illumina’s sequencing-by-synthesis (SBS) approach, these libraries are sequenced, generating vast quantities of short-read data at high throughput. Compared with traditional Sanger sequencing, Illumina sequencing offers superior capabilities, including high-throughput scalability, cost effectiveness, and comprehensive coverage of mRNA. Illumina sequencing is especially known for its precision and reproducibility, yielding data suitable for analyzing gene expression profiles, identifying novel transcripts, and investigating alternative splicing events. Bioinformatics pipelines process these reads, generating consensus sequences that align with specific mRNA targets, followed by downstream analyses tailored to research objectives, such as differential expression analysis, transcript discovery, and functional annotation. Although Illumina sequencing is often limited by shorter read lengths (typically 50–3000 bp), it remains one of the most cost-effective and scalable options for transcriptomic studies [110].

**Figure 10:**
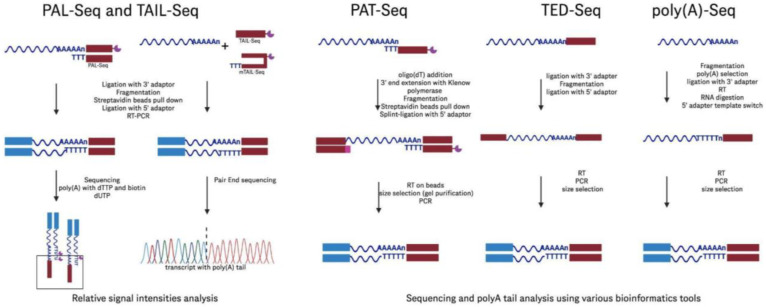
Overview of Illumina-based methods for sequencing RNA 3′ ends. Solid red blocks represent barcode sequences, which may include or exclude an oligo(dT) sequence. Purple regions indicate 5′ barcodes, while the 3/4 purple semicircles represent biotin modifications attached to the 3′ adapter. Poly(A) tail measurement techniques have evolved significantly since the introduction of PAL-Seq and TAIL-Seq (**left panel**). Despite advancements, challenges remain, particularly in balancing read length, adapter compatibility, and computational processing. Newer methods, including poly(A)-Seq and TED-Seq (**right panels**), improve upon earlier techniques but require careful consideration of sequencing depth, sample recovery, and bioinformatic tools. Each method presents trade-offs in resolution, efficiency, and applicability, underscoring the need for continued development in mRNA tail analysis.

Illumina-based sequencing techniques not only can be utilized for determining mRNA identity but have also significantly advanced to offer detailed methods for detecting poly(A) tail lengths in mRNA vaccines. Here, we focus on detailing several Illumina-based methods for detecting poly(A) tails and their potential applications in enhancing our understanding of mRNA vaccine quality and efficacy, while acknowledging the historical diversity of approaches in this field [11]. PAL-Seq [92] and TAIL-Seq [93] were among the early methods, developed in 2014, to offer genome-wide poly(A) tail profiling on Illumina platforms (Figure 10). PAL-Seq utilizes a biotinylated dUTP to label poly(A) tails, allowing transcript identification via fluorochrome-conjugated streptavidin. Despite its effectiveness, its reliance on outdated technology (the now-obsolete Illumina Genome Analyzer) limits its utility. TAIL-Seq, on the other hand, is compatible with modern Illumina sequencers like HiSeq and MiSeq and incorporates advanced fluorescence quantification for poly(A) tail length estimation but requires tailored control software to store intermediate fluorescence intensity files. Notably, a modified version of TAIL-Seq (mRNA TAIL-seq [mTAIL-seq]) [94] enhanced ligation efficiency by incorporating a hairpin oligonucleotide adapter, enabling use on smaller sequencers like MiSeq with reduced sequencing depth (Figure 10). Poly(A)-Seq incorporates an oligo(dT) selection and ligation of 3′ adapters post-fragmentation, improving poly(A) tail length estimates for tails longer than those detected by TAIL-Seq [93]. Using 300 nt single reads on the NextSeq 500, poly(A)-Seq captures longer tails (up to ~150 nts), although size selection biases toward longer tails; pA-finder, the associated bioinformatics pipeline, filters reads based on adenosine content, offering enhanced resolution at longer lengths but excluding tails shorter than 10 nts, leading to data loss in very short poly(A) tail populations. Each of these methods leverages different protocols for 3′ adapter ligation, sequencing, and bioinformatic processing to provide nucleotide-resolution measurements with varied compatibility across Illumina sequencers (Figure 10).

In addition to the 3′ ligation-based methods discussed, several additional Illumina-based techniques have been developed to further enhance poly(A) tail analysis in mRNA. PAT-Seq [96] improves poly(A) tail measurement by extending the RNA’s 3′ end with Klenow DNA polymerase, using directional sequencing to mitigate issues from homopolymer stretches. Of note, PAT-Seq offers correlations with PAL-Seq data, although it is limited by its ability to detect only shorter poly(A) tails (<80 nts) due to 100 bp read lengths. Longer read protocols have improved tail detection up to 150 bp, demonstrating effective use in differential expression analysis [96]. TED-Seq [99] applies standard Illumina sequencing by mapping 3′ cleavage and polyadenylation sites by calculating the distance from mapped 5′ ends to 3′ poly(A) tails, normalized against known spike-ins. This cost-effective approach, requiring precise size selection, can achieve a 10 bp resolution but lacks quantitative robustness due to variable recovery rates across laboratories. Specialized techniques have also been developed to address specific challenges in poly(A) tail measurement. One such method is circTAIL-Seq [111], which integrates circular reverse transcription PCR (circRT-PCR) with next-generation sequencing to measure poly(A) tails in low-abundance or mitochondrial RNAs. This technique is particularly effective for analyzing shorter and heterogeneous tails, making it well suited for mitochondrial RNAs that often exhibit unique tail compositions. However, circTAIL-Seq has not been validated for the longer poly(A) tails commonly observed in mRNAs, limiting its use in certain applications. Despite this limitation, the method provides valuable insights into the tail composition and dynamics of specialized RNA populations, contributing to a more comprehensive understanding of RNA biology (Figure 10).

When evaluating mRNA vaccine quality, a comprehensive understanding of all tail modifications, including non-poly(A) tails, can provide insights into the tailing efficiency and thus the mRNA’s stability, degradation rates, and overall effectiveness. Additionally, some mRNA constructs might incorporate non-poly(A) tails to enhance stability or control translation rates differently. In such cases, QC processes should include methods that can accurately detect and characterize these tail types. For non-poly(A) tails, we highlight two such examples: EnD-Seq [97] and 3′RACE-Seq [98]. EnD-Seq targets short poly(A) tails and non-polyadenylated RNAs by ligating a 3′ adapter to preserve termini. This protocol, paired with the AppEnD software, has been primarily used in histone mRNA studies as the mRNAs encoding canonical histone genes typically end in a conserved stem-loop structure instead of a poly(A) tail [112], demonstrating efficacy for detecting short tails (up to 15 nt). Similarly, 3′RACE-Seq enables targeted analysis of specific transcripts, valuable for studying ncRNA termini and end modifications in model systems [98], which works by using reverse transcription followed by amplification with a specific primer that targets the RNA of interest, allowing for precise identification and characterization of the 3′ ends of the transcripts, even in complex mixtures. These techniques can be utilized to detect and characterize non-polyadenylated RNA tails, providing valuable insights into mRNA modifications that can impact vaccine stability and function.

Taken together, these methods can contribute to the field of mRNA vaccine analysis by providing identity and precise measurements of poly(A) tail lengths, as well as non-poly(A) tails, which are crucial for determining mRNA stability and translation efficiency. This detailed profiling capability enhances our understanding of the quality and functionality of mRNA vaccines, ultimately aiding in the development and optimization of vaccine candidates for better efficacy and safety.

Beyond sequence identity and poly(A) approaches, Ribo-seq, or ribosome profiling, is a powerful technique that provides insights into translation by capturing snapshots of ribosomes on mRNA transcripts [100,101]. The Ribo-seq workflow involves isolating ribosome-protected mRNA fragments (RPFs), purifying mRNA sequences protected by the bound ribosomes, followed by library preparation and Illumina sequencing. In the context of mRNA vaccine development, Ribo-seq can be utilized to monitor the translation efficiency of vaccine mRNAs, ensuring that they are effectively translated into proteins once delivered into host cells. This technique allows researchers to map the exact location of ribosomes on mRNA molecules, providing a detailed view of which portions of the mRNA are being actively translated. By applying Ribo-seq, developers of mRNA vaccines can optimize the coding sequences to enhance protein production [113]. Additionally, Ribo-seq can help identify potential translation bottlenecks, enabling further refinement of mRNA vaccine constructs for improved efficacy and safety. Although it is still early days, Ribo-seq data are increasingly being integrated with artificial intelligence and machine learning models to predict sequence translation efficiency and identify potential liabilities, paving the way for even more precise mRNA vaccine design [114,115]. This makes Ribo-seq an invaluable and complementary tool in the comprehensive analysis and optimization of mRNA vaccine candidates.

### 5.3. PacBio Sequencing for Long-Read Accuracy

**Figure 11:**
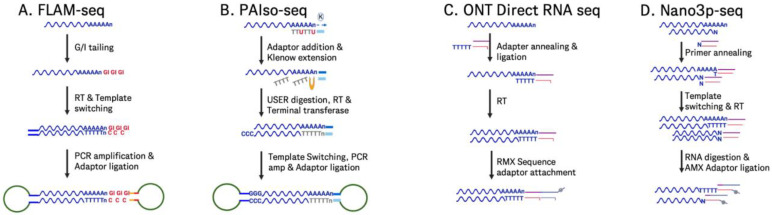
Overview of long-read RNA sequencing methods for capturing full-length RNA, including poly(A) sequences. Panels (**A**,**B**) depict methods utilizing the PacBio sequencer with green SMRTbell adapters. Panels (**C**,**D**) illustrate methods employing the Oxford Nanopore sequencer. In panel (**A**), the orange region represents the unique molecular identifier (UMI).

PacBio’s single-molecule, real-time (SMRT) sequencing has transformed transcriptomic analyses, allowing for full-length sequencing of mRNA isoforms. Unlike Illumina’s short-read sequencing-by-synthesis approach, PacBio utilizes circular consensus sequencing (CCS) technology (Figure 11), in which a single molecule is read multiple times, producing long, high-fidelity reads with accuracies reaching up to 99.8% [106]. This makes it well suited for applications like Isoform Sequencing (Iso-Seq), PacBio’s protocol for generating full-length cDNA reads without assembly. Iso-Seq’s ability to handle homopolymers is particularly valuable for studying poly(A) tails as it provides isoform-specific tail length and composition data, even within complex or repetitive regions. Here, we detail several notable PacBio-based protocols published in recent years, full-length mRNA sequencing (FLAM-Seq [107]), Poly(A) inclusive RNA isoform sequencing (PAIso-Seq [108]), and single-molecule Poly(A) tail sequencing (SM-PAT [109]); they are among the standout methodologies that offer unique advantages in these areas, particularly for mRNA vaccine analysis.

FLAM-Seq is designed for profiling poly(A) tails by capturing the entire length of polyadenylated RNA (Figure 11). This entails isolating the poly(A)+ fraction of RNA, followed by G/I tailing at the 3′ end. Reverse transcription is carried out with template switching to incorporate necessary adapters, generating double-stranded cDNA for PacBio sequencing [107]. This method is advantageous for studies requiring isoform-specific information as it provides long, highly accurate reads that capture poly(A) tail lengths and internal composition. For mRNA vaccines, this means ensuring that the poly(A) tail is of the correct length and composition, which is essential for the stability and translation efficiency of the vaccine mRNA. However, PCR amplification bias is a consideration, as it may impact the uniformity of tail lengths in the final data. Additionally, the cost of FLAM-Seq limits its widespread use, especially for high-throughput applications.

PAIso-Seq similarly profiles poly(A) tails but introduces a template oligonucleotide at the 3′ end to avoid direct poly(A)+ RNA selection (Figure 11). This method bypasses biases associated with oligo(dT) selection, increasing sensitivity and allowing analysis from minimal starting material, even single cells. The protocol involves oligonucleotide-guided 3′ end extension, followed by reverse transcription and PacBio sequencing [108]. PAIso-Seq offers long reads and high-accuracy isoform-specific data with minimal starting material requirements, although its reliance on the Klenow polymerase can sometimes introduce artifacts in poly(A) tail measurements. Despite its higher cost, PAIso-Seq’s reduced RNA requirements and bias reduction make it ideal for applications where sample material is limited or accuracy is paramount.

SM-PAT involves selecting polyadenylated mRNAs with oligo(dT) and adding a 3′ adapter through splint ligation. The protocol includes reverse transcription, second-strand synthesis, and PCR amplification before sequencing. SM-PAT distinguishes itself by enabling full-length poly(A) tail profiling, including detailed internal tail composition and isoform-specific tail information. It has been used to study the role of proteins such as LARP4 in regulating poly(A) tail length and stability, showing high correlation with Northern blot data [109]. However, similar to other protocols, SM-PAT requires PCR, which may lead to amplification bias, and its cost impacts accessibility for routine poly(A) tail analysis.

The PacBio-based protocols detailed here, FLAM-Seq, PAIso-Seq, and SM-PAT, offer significant advancements and potential for mRNA vaccine profiling, providing long reads and high accuracy, as well as comprehensive poly(A) tail profiling. Compared with Illumina, PacBio’s long-read capability and sensitivity to homopolymer regions offer a distinct advantage in profiling the full length and composition of poly(A) tails. While Illumina remains a popular choice due to its cost-effectiveness and extensive protocol and instrument infrastructure, its shorter reads and sequencing-by-synthesis approach are less suited for full-length mRNA and homopolymer analysis. Overall, PacBio’s SMRT technology is better equipped for studies where accuracy and detailed poly(A) tail characterization are priorities, despite its higher cost and limited availability.

### 5.4. Oxford Nanopore Sequencing for Versatile Long-Read Solutions

The rapid development and deployment of mRNA vaccines have underscored the critical need for precise and comprehensive analytical tools. Oxford Nanopore Technologies (ONT) has emerged as a pivotal player in this domain, offering advanced long-read sequencing capabilities that provide unparalleled insights into the structure and function of mRNA vaccines. The ability to directly sequence RNA molecules, along with template plasmid DNA, is particularly beneficial for assessing critical quality attributes (CQAs) of mRNA vaccines. These capabilities allow researchers to precisely evaluate poly(A) tail lengths, detect post-transcriptional modifications, identify off-target RNA species, and uncover potential contaminants in template plasmid sequences, which are key factors influencing the stability, efficacy, and safety of mRNA vaccines.

Several innovative methods leveraging ONT platforms have been developed for these purposes. VAX-seq [102] integrates all three sequencing methods offered by ONT (DNA, cDNA, and RNA) to analyze CQAs of vaccines. Nano3P-seq [116], an enhancement of existing ONT approaches, identifies aborted transcripts and mRNA fragments lacking poly(A) tails. FLEP-seq [105] enables the detection of RNA polymerase positioning, splicing status, and poly(A) tail lengths, providing insights into transcriptional dynamics. Additionally, nanoSHAPE [103] is utilized to detect RNA modifications [91], adding another layer of detail to the analysis.

VAX-seq [102] has emerged recently as a comprehensive workflow demonstrating how nanopore long-read sequencing can accurately assess mRNA quality attributes, such as sequence identity, integrity, poly(A) estimation, and contamination. VAX-seq employs Oxford Nanopore Technologies’ long-read sequencing to evaluate mRNA vaccine quality by focusing on poly(A) tail length and the integrity of the mRNA construct. In a study involving an enhanced green fluorescent protein mRNA vaccine, VAX-seq effectively anchored a reverse transcriptase primer to the poly(A) tail’s 3′ terminus, enabling complete sequencing and precise tail length measurement after correcting for systematic deletion errors using Tailfindr software. Unlike Illumina’s short-read sequencing, which encounters challenges with poly(A) tail analysis due to misalignment errors, VAX-seq successfully distinguishes full-length mRNA from truncated molecules. The method revealed that nearly 58.2% of reads encompassed the full mRNA length, while the remainder showed 3′ or 5′ truncation, likely due to transcriptional or degradation processes. VAX-seq demonstrated high reproducibility and could identify contaminating RNA, with approximately 93% of sequences aligning to the on-target mRNA. This approach highlights its utility in providing detailed insights into mRNA vaccine quality, particularly in assessing poly(A) tail length and identifying potential off-target effects. As sequencing technologies continue to advance alongside bioinformatics tools, more integrated workflows are expected to emerge, offering comprehensive insights into mRNA-based therapeutics.

Traditionally, nanopore sequencing has concentrated on analyzing polyadenylated RNA through either cDNA or direct RNA sequencing techniques, which has constrained its capacity to identify non-polyadenylated transcripts. Nano3P-seq [116] overcomes this limitation by offering a nanopore cDNA sequencing approach capable of capturing both polyadenylated and non-polyadenylated RNA at the single-molecule level (Figure 11). Utilizing a template-switching method, Nano3P-seq delivers simultaneous insights into RNA abundance, Poly(A) tail structure, and length variations. Nano3P-seq modifies the ONT direct cDNA sequencing protocol by integrating TGIRT template switching, which facilitates sequencing from the RNA’s 3′ end. This method allows for precise quantification of RNA levels and Poly(A) tail lengths across a wide variety of RNA types, encompassing both coding and non-coding RNAs. In studies involving mouse and zebrafish models, Nano3P-seq successfully identified Poly(A) tails in mRNA, long non-coding RNAs (lncRNAs), and 16S mitochondrial ribosomal RNA, demonstrating its extensive applicability. Overall, Nano3P-seq stands as a powerful potential tool for the comprehensive analysis of mRNA vaccines, offering robust measurements of poly(A) tail length and composition with minimal bias. This capability is crucial for ensuring that mRNA vaccines are designed to optimize stability and translation efficiency, ultimately enhancing their efficacy and safety.

FLEP-seq [105] (full-length extended polyadenylation sequencing) is a powerful technique that converts nascent RNA molecules into full-length cDNA for sequencing, providing comprehensive insights into RNA biology. The method involves depleting ribosomal RNA (rRNA) and transfer RNA (tRNA) to enrich for messenger RNA (mRNA) and other RNA species, followed by the ligation of a universal DNA adapter to the 3′ end of the RNA molecules. Reverse transcription (RT) with template switching then generates full-length cDNA, preserving complete RNA sequences, including poly(A) tails. After PCR amplification, the cDNA is sequenced on the ONT or Pacbio platform, producing long reads that span entire cDNA molecules. This approach enables the simultaneous detection of key RNA features, such as RNA polymerase II positioning, splicing status, polyadenylation sites, and poly(A) tail lengths.

Oxford Nanopore Technologies sequencing can be effectively combined with chemical probing methods to analyze RNA structure, particularly through the innovative nanoSHAPE [103] technique. The nanoSHAPE method is an iteration of SHAPE-MaP (selective 2′-hydroxyl acylation analyzed by primer extension and mutational profiling) [103], providing full-length structural insights at single-molecule resolution using nanopore sequencing. By using chemical reagents (2′-hydroxyl reactive compounds) that modify RNA at flexible, unpaired regions, nanoSHAPE allows researchers to map these structural features directly through sequencing. The RNA of interest, after chemical modification, is sequenced alongside an unmodified control RNA, and variations in translocation rates during sequencing are analyzed to computationally infer secondary structures. In the context of mRNA vaccines, understanding RNA structure is important because it can influence the stability and translation efficiency of the mRNA, ultimately affecting the vaccine’s efficacy. Additionally, certain structures might protect the mRNA from degradation, prolonging its presence in the cell and enhancing protein production [117,118]. NanoSHAPE’s ability to provide high-resolution structural information complements other sequencing methods by offering insights beyond sequence data, making it a valuable tool in the comprehensive analysis and optimization of mRNA vaccines.

ONT offers direct RNA sequencing capabilities that circumvent the need for cDNA synthesis, which is particularly advantageous in preserving the native RNA molecule. This capability is crucial for mRNA vaccine development as it allows for the confirmation of mRNA identity and the detection of nucleoside modifications, providing a broader understanding of the mRNA sequence and its underlying structure. Unlike traditional sequencing methods that require reverse transcription and amplification, ONT’s approach enables the analysis of RNA in its native state. This direct method is especially beneficial for the site-specific detection of important modified nucleosides, such as pseudouridine and N1-methylpseudouridine (m1Ψ) [119,120], which play critical roles in enhancing mRNA stability, translation efficiency, and immunogenicity, all these key factors determining the effectiveness of a vaccine [121,122]. However, a known limitation of direct RNA sequencing is its higher error rates compared with cDNA sequencing. That said, there is no doubt that these error rates will continue to improve with advancements in sequencing technology and bioinformatics. Over the past few years, the error rates for nanopore sequencing have seen significant improvements due to the development of better algorithms for base calling and sequence correction with a species-dependent accuracy ranging from 87% to 92% [123]. Despite this, the ability to directly detect and characterize RNA modifications offers invaluable insights into the design and optimization of mRNA vaccines, ensuring they are both effective and stable.

As mRNA vaccine research progresses, advanced sequencing technologies offer immense potential to transform vaccine development by providing critical insights into mRNA design, quality, and function. Illumina, PacBio, and ONT each offer distinct advantages that cater to different facets of mRNA analysis, from high-throughput short-read sequencing to comprehensive long-read and direct RNA sequencing. By providing detailed insights into mRNA structure, poly(A) tail dynamics, and nucleotide modifications, these platforms empower researchers to fine-tune vaccine candidates, improving their stability; translation efficiency; and, ultimately, their immunogenic potential. As new sequencing methods continue to emerge, they will undoubtedly play a critical role in advancing our understanding of mRNA vaccines, paving the way for more effective and safer vaccines in the future. Through continued innovation and application of these cutting-edge technologies, the scientific community is well positioned to tackle the challenges of mRNA vaccine development and meet the ever-growing demands of global health.

## 6. Functionality Testing

Beyond physicochemical techniques for characterizing specific quality attributes, it is also critical to have assays that assess the overall functionality of mRNA. Functionality testing evaluates the combination of all the analytical characteristics of the mRNA, such as capping efficiency, poly(A) tail, dsRNA content, and other structural features that ensure that the synthesized mRNA can be efficiently translated into a functional protein. These assays typically evaluate translation efficiency, often measured as the yield and/or quality of the encoded protein product. There is a wide variety of assays to measure mRNA translation efficiency, ranging from cell-free systems utilizing recombinant proteins to more complex cell-based assays.

The ease of cell-free systems such as in vitro translation assays make them attractive. These assays are also agnostic to the mRNA’s role as they only test the translation and not the downstream function of the protein encoded by the mRNA. They use cellular lysates like Rabbit Reticulocyte Lysate (RRL), Wheat Germ Extract (WGE), or HeLa Cell Lysate, which include all components necessary for translation from a cellular context. This can make them more biologically relevant than fully recombinant systems such as the PURE system. Translation efficiency can be monitored by incorporating luciferase reporter genes into the mRNA sequence [124], measuring luminescence as a direct and quantitative readout of translation activity. Alternatively, Western blot analysis can be used to assess the production of specific sequences without a reporter gene, tracking protein expression levels. This involves resolving proteins on an SDS-PAGE gel, transferring them onto a membrane, and using antibodies for detection. While Western blots require specific antibodies, incorporating fluorescently labeled amino acids allows the visualization of all protein products without antibodies. This method allows researchers to determine the abundance of the target protein relative to any prematurely terminated products.

While cell-free systems are robust and relatively simple, given the diverse applications of mRNA—ranging from vaccines, immunotherapies, protein replacement therapies, and genome editing—ideally, functional assays directly assess the desired mechanism of action of each mRNA-based application. Furthermore, it is often most informative to test the mRNA in conjunction with its delivery vehicle as this provides a more comprehensive understanding of its functionality in a biological system. Consequently, using cellular assays is generally preferred as they allow for a more accurate assessment of mRNA expression and functionality within the context of its intended delivery and application.

For mRNA vaccines the functionality is defined as the faithful translation of a single or series of antigens by the ribosomes. This is best done in the context of a cell with an mRNA delivered in its clinical formulation. For example, Patel et al. reported the development of an imaging-based in vitro assay capable of quantifying transgene protein expression efficiency, which is useful for measuring LNP-encapsulated mRNA vaccine potency, efficacy, and stability [125]. More recently, Q. Stiving et al. developed a platform-based, antibody-free method using cell-free translation and LC-MS/MS to detect, characterize, and provide relative quantification of antigen proteins translated from mRNA vaccine (mRNA encoding different antigen variants) [126]. The sensitivity and specificity of the method were successfully demonstrated by identifying all six translated proteins and their relative abundances in a dose-dependent manner following the transfection of human cells with a hexavalent mRNA mixture encapsulated in LNPs.

The primary function of an mRNA is to carry the code needed to direct the translation of a specific protein. This can be assessed independently of that protein’s activity. However, testing the functionality of the encoded protein is also critical as it verifies whether the mRNA sequence is adequately designed to produce the intended protein capable of performing its specific function. This adds complexity to the analysis as each protein requires an assay designed to evaluate its specific function. Genome editing using mRNAs encoding TALEN, ZFN, and Cas9 is a perfect example where the approach is to test the functionality of the genome-engineering enzyme [127]. This involves using mRNA that expresses the enzyme in gene-editing experiments. Scientists transfect cells with Cas9 mRNA and specific guide RNAs (gRNAs) that direct Cas9 to targeted genomic loci. Gene-editing efficiency is then measured using techniques such as antibody detection of the targeted protein, the T7 endonuclease assay [26], next-generation sequencing [128], or reporter assays [129]. By assessing the frequency and accuracy of the introduced modifications, it is possible to determine the functional activity of Cas9 produced by the mRNA. This method is important for applications in gene and cell therapy. The efficiency and precision of gene modifications are directly correlated with the ability of the mRNA to effectively express the Cas9 protein, making this assay essential for evaluating the mRNA used in gene-editing applications.

Functionality assays can also be developed to test for impurities that may be generated during the in vitro transcription manufacturing process. For example, murine cells are highly sensitive to dsRNA impurities due to the presence of certain sensors (e.g., RIG-I-like receptors, protein kinase R, oligoadenylate synthases, Toll-like receptors and NOD-, and LRR- and pyrin domain-containing) [130]. Therefore, a valuable assay for detecting the level of dsRNA produced in an IVT mRNA preparation is to deliver the RNA to mouse cells in vitro and measure cytokine production (e.g., TNF-α and IL-6). A recent study used this approach to evaluate various engineered T7 RNA polymerases, with the goal of reducing the synthesis of dsRNA in IVT preparations [51]. The level of protein expression was analyzed using flow cytometry, while the innate immune response was determined by measuring the expression of known activation markers on the cell surface and the level of cytokine release into the supernatant using Luminex analysis. High immune responses associated with reduced mRNA translation suggest the presence of immunogenic contaminants or problematic mRNA sequences. This assessment ensures that IVT mRNA does not induce unintended immune responses, which is essential for therapeutic applications. This approach is crucial for ensuring the safety and non-immunogenicity of therapeutic mRNAs, especially for clinical applications where immune activation must be minimized.

## 7. Overview of Analytical Technologies for mRNA Characterization

This review has discussed several analytical technologies, each offering distinct advantages and limitations for characterizing mRNA-based therapeutics. Considerable effort has been invested in developing new analytical tools to characterize the diverse critical quality attributes of mRNA-based therapeutics. Since no single assay can comprehensively characterize the entire set of CQAs, multiple approaches, including CE, chromatography, mass spectrometry, mass photometry, and new sequencing technologies, are employed to cover the characterization of CQAs. Table 5 provides an overview of the discussed analytical methods, highlighting their respective advantages and limitations. Selecting the appropriate method depends on the specific analysis requirements, ensuring comprehensive and precise characterization of mRNA therapeutics.

## 8. Conclusions

The COVID-19 pandemic has catalyzed rapid advancements in RNA-based therapies, exemplified by the successful development and global deployment of mRNA vaccines. These achievements have positioned mRNA-based drugs as a transformative therapeutic class, with promising applications in cancer treatment, including therapeutic vaccines, monoclonal antibodies, immunomodulatory drugs, and cell therapies. The clinical efficacy and recent approvals of mRNA products underscore their potential to revolutionize healthcare.

Advances in analytical technologies have provided researchers with powerful tools to characterize quality attributes of RNA-based products. These tools are instrumental for process optimization; formulation development; and establishing robust methods for quality control, batch release, and stability testing. Innovations in capillary electrophoresis, such as new CGE kits or microCE systems, have improved the resolution and throughput of mRNA purity analyses. Similarly, recent advances in HPLC technologies, including specialized RNA-dedicated columns and optimized mobile phase gradients, have enabled enhanced separation performance across various chromatographic modes like AEX, SEC, and IP-RP LC. These techniques collectively enable detailed profiling of mRNA impurities and facilitate orthogonal assessment of specific quality attributes. Mass spectrometry technologies, particularly high-resolution Orbitrap analyzers and UHMR instruments, now allow precise characterization of mRNA molecules. Coupled with improved sample preparation strategies and LC-MS/MS workflows, they offer high-resolution analyses of complex oligonucleotide mixtures, enhancing sequence coverage, with a detailed evaluation of sequence composition and impurity profiles at the nucleotide level. Emerging next-generation sequencing technologies, encompassing both short-read and long-read platforms, further empower researchers to evaluate critical mRNA attributes such as sequence identity, structural integrity, purity, poly(A) tail length, and post-transcriptional modifications. Together, these technologies provide comprehensive insights into mRNA quality and functionality.

Although the development of functional assays for RNA-based products remains a challenge due to the need to reflect the mode of action and their dependence on a combination of CQAs (e.g., 5′ cap, 3′ poly(A) tail, integrity, and dsRNA content), the integration of advanced analytical methods enables the consistent production of high-quality, safe, and effective mRNA-based therapies. By leveraging these innovations, RNA-based drugs can meet stringent clinical and commercial standards, paving the way for their widespread application in modern medicine.

## Figures and Tables

**Table 1:** CE-based applications for mRNA characterization.

RNA Samples	Analytical Method Conditions					Target QA	General Comments	Ref.
	CE Mode	CE Capillary/Gel Type	Background Electrolyte (BGE)	Detection Mode	Separation Time			
- mRNA-LNP (~2000 nts)	mCE	RNA labchips	RNA reagent kits (Catalog# CLS960010)	LIF	70 s	Purity and integrity	Development and optimization of a purity and integrity assay for mRNA-based vaccines encapsulated in LNPs.	[28]
- eGFP (996 nts) - FLuc (1909 nts) - β-Gal (3420 nts)	mCE	RNA labchips	RNA reagent kits (Catalog# CLS960010)	LIF	70 s	Purity and aggregate contents	mCE used in non-denaturing conditions (heated at 70 °C for 10 min) to determine the percentage of covalent and non-covalent aggregates.	[30]
- eGFP (996 nts) - FLuc (1909 nts) - β-Gal (3420 nts)	CGE	Fused-silica capillary length of 60 cm (50 μm i.d.)	SCIEX RNA 9000 Purity and Integrity kit	LIF	100 min	Purity and aggregate contents	CGE used in denaturing conditions to monitor covalent aggregates.	[30]
- eGFP (996 nts) - Luciferase (2000 nts) - Cas9 (4500 nts)	CGE	Fused-silica capillary length of 30 cm (100 μm i.d.)	Tris-Borate with urea and methylcellulose	UV	40 min	Poly(A) tail length	CGE is used for the determination of the poly(A) tail length, with a resolution comparable with the IP-RP LC method.	[29]
RNA marker (100 to 10,000 nts)	CGE	Fused-silica capillary length of 15 cm (75 μm i.d.)	PEG polymer, TBE, 4 M urea	LIF	10 min	N/A	Investigation of the separation of RNA fragments in PEG and PEO solutions.	[31]
- EPO (859 nts)	CGE	Fused-silica capillary length of 72 cm (50 μm i.d.)	Tris-Borate-EDTA HEPES buffer (pH 7.5) with polymer solution (PVP and glycerol)	UV	130 min	Purity	Design a flexible, multi-objective CGE method for analysis of modified mRNA by focusing on the components of the low-viscous polymer matrix.	[32]
- Single-stranded RNA size marker (size ranging from 281 to 6583 nts)	CGE	Fused-silica capillary length of 21.5 cm (50 μm i.d.)	Tris-Borate-EDTA buffer with urea and HEC as the polymer solution	UV	10 min or 20 min	N/A	Investigation of electrophoretic separation of large RNA (over 6000 nts) in dilute and semidilute polymer matrices.	[33,34]
BNT162b2 mRNA (active substance)	CGE	Fused-silica capillary (50 μm i.d.)	Agilent RNA Analysis Kit	LIF	60 min	Purity	Analysis of short impurity species to be 5’-end BNT162b2 fragments generated from premature transcription stop during the IVT reaction.	[24]
RNA marker (100 to 10,000 nts)	CGE	Fused-silica capillary length of 9 cm (75 μm i.d.)	10X TBE, HEC	LIF	8 min	N/A	Investigation of the effect of MW of HEC on the separation performance of long RNA. HEC favors the separation of short RNA fragments (<1000 nts).	[35]
- RNA size marker (size ranging from 200 to 6000 nts)	CGE	Fused-silica and PVA-coated capillary length of 56 cm (50 μm i.d.)	A stock formamide buffer (pH 6.0) prepared in formamide or water and containing MES and EDTA with or without urea	UV	20 and 50 min	N/A	CGE method using high MW polymers and formamide, enhancing the resolution for mRNAs by approximately six-fold compared with standard aqueous CGE methods.	[27]
- eGFP (996 nts) - Luciferase (2000 nts) - Cas9 (4500 nts)	CGE	Fused-silica capillary length of 30 cm (100 μm i.d.)	ssDNA 100-R Gel reconstituted in Tris-Borate-7 M urea buffer	UV	28, 32, and 40 min for eGFP, Luciferase, and Cas9, respectively	Poly(A) tail lengh	CGE method having the same resolution as an LC-MS method for the characterization of the poly(A) tail length.	[29]
- eGFP (996 nts) - Ovalbumine (1437 nts) - Luciferase (1929 nts) - Cas9 (4521 nts)	CGE	(1) Agilent 2100 Bioanalyzer along with the RNA nano chips (2) Revvity Labchip XII + HT DNA 5K/RNA/charge variant assay LabChip (3) Sciex PA800 Plus, fused-silica capillary length of 30 cm (50 μm i.d.) (4) Agilent Fragment Analyzer, using a 33 cm capillary array	(1) Gel-dye matrix—RNA 6000 Nano Reagent Kit (2) Gel reagent—RNA Reagent kit (3) RNA 9000 Purity & Integrity Kit used as a separation buffer (4) HS RNA kit used for the purity analysis	(1) LIF (2) LIF (3) LIF (4) LIF	(1) 70 s (2) 70 s (3) 22 min (4) 40 min	Integrity, purity	Sciex RNA 9000 Purity and Integrity Kit provides the highest selectivity and resolving power for characterization of mRNA. Agilent 600 Nano Kit, Revvity RNA Reagent Kit, and Agilent HS RNA Kit offer faster analysis times, making them more suitable for high-throughput and screening applications.	[36]
tRNAPhe (75 nt) and HIV-1 5′-UTR (364 nt)	CZE	High-resolution bare glass chips (HRB)	Mix of ammonium acetate with H_2_O+ 25% IPA	MS	5 min	Sequence confirmation and impurities	CZE-MSl for the bottom-up characterization of nucleic acids, using microfluidic devices that combine both capillary and transmitter in the same chip.	[37]
- Modified linear GLuc mRNA - Modified hEpo mRNA - GLuc APIE CVB3 pAC (circRNA)	AGE	Agarose E- EX gel (2%)	Tris-acetate-EDTA buffer with formamide	Bands are visualized using blue light transillumination	80 min	Purity	AGE for the separation of circular splicing products (i.e., linear precursor molecules, nicked circles, splicing intermediates, and excised introns).	[38,39]

AGE, agarose gel electrophoresis; β-Gal, beta galactosidase; CGE, capillary gel electrophoresis; CZE, capillary zone electrophoresis; circRNA, circular RNA; EPO, erythropoietin; eGFP, enhanced Green Fluorescent Protein; IPA, isopropanol; FLuc, firefly luciferase; HEC, hydroxyethylcellulose; LIF, laser-induced fluorescence; LNPs, lipid nanoparticles; mCE, microcapillary electrophoresis; MW, molecule weight; N/A, not applicable; PEG, polyethylene glycol; PEO, polyethylene oxide; QA, quality attribute.

**Table 2:** HPLC-based applications for intact mRNA analysis.

RNA Samples	Analytical Method Conditions				Target QA	General Comments	Ref.
	LC Mode	LC Column	Mobile Phase, Column Temperature	Detector			
mRNA samples produced from eGFP, C-Spike and NLuc plasmid DNA templates	AEX	DNAPac PA200 (50 mm × 2.1 mm)	MPA: 10 mM NaOH MPB: 2 M NaCl, 25 °C	UV	Method to separate IVT components (e.g., NTPs, Cap analogue, plasmid DNA, and mRNA)	In process IVT mRNA impurities.	[44]
Short oligonucleotides and EGFP (996 nts) mRNA	AEX	DNAPac PA200 (50 mm × 2.1 mm)	Neutral pH—sodium chloride or sodium perchlorate salt gradient—60 °C, or high pH (pH = 12)—salt gradient separation—10 °C	UV	Purity—degradation RNA products	AEX using denaturing conditions.	[45]
- Cas9 (4500 nts) - EPO (859 nts)	AEX (IPAX)	Protein-Pak Hi Res Q strong anion exchange (50 × 2.1 mm, 5 µm)	25 mM HEPES and TRIS buffers (pH 7.5–8) and 1–3 M TMAC gradients, up to 60 °C	UV	Purity—degradation RNA products	Using a gradient of weak ion-pairing cations (e.g., TMAC) to provide different recovery and selectivity effects.	[46]
- EPO (859 nts) - β-FLuc (~2000 nts)	SEC	ACQUITY Premier protein SEC (250 Å, 1.7 μm, 4.6 × 150 mm)	0.1 M phosphate buffer, pH 8, 25 °C	UV	Poly(A) tail length	Average poly(A) tail length.	[10]
- EGFP (996 nts) - Cre (1350 nts)	SEC	Ultrawide pore prototype SEC (4.6 mm I.D. x 300, 3.0 μm, 1275 Å) column—silica-based packing material modified with an OH-PEG bonding	50 mM Tris and 200 mM potassium chloride (pH adjusted to 7.5, 25 °C)	UV, RI, and MALS	Purity—short fragments and aggregates	Use of prototype, low adsorption ultrawide pore SEC columns.	[47]
Various mRNA samples (996–4521 nt), including modifications (5moU), stressed samples, and from different suppliers	SEC	GTxResolve Premier BEH™ SEC (450 Å, 2.5 µm, 150 × 4.6 mm) GTxResolve Premier SEC 1000 (1000 Å, 3 µm, 150 × 4.6 mm and 300 × 4.6 mm) Ultrawide pore prototype SEC (2500 Å, 5 µm, 150 and 300 × 4.6 mm)	50 mM Tris buffer, pH 7.5, 200 mM KCl and 10 mM MgCl_2_. 25 °C	UV	Purity—short fragments and aggregates	Use of ultrawide pore columns Addition of 10 mM MgCl_2_ to improve chromatographic resolution and/or preserve mRNA from confounding effects (in some cases).	[48]
FLuc (~2000 nts)	SEC	Agilent Bio SEC-5 (4.6 × 300 mm, 5 μm) with varying pore size (300 Å, 1000 Å and 2000 Å)	100 mM phosphate, pH 7.0, 25 °C	UV, RI, and MALS	Purity—MW, short fragments, and aggregates	Optimization of LC conditions on the separation performance and structural integrity of mRNAs.	[49]
- eGFP (996 nts) - FLuc (1909 nts) - β-Gal (3420 nts)	SEC	SRT(R) SEC-1000 gel (4.6 mm I.D. × 300 mm, 5 μm particle size, 1000 Å, stainless steel)	100 mM Tris–HCl and 300 mM NaCl at pH 7.5, 25 °C	UV	Purity—MW, short fragments, and aggregates	Level of mRNA aggregates can be significantly reduced after a heating step. Limitation of SEC mode for mRNA aggregates.	[30]
- Large RNA, up to 1000 nts - eGFP (996 nts)	IP-RP	DNAPac RP (150 mm × 2.1 mm i.d.)	MPA: 100 mM TEA in H_2_O MPB: 100 mM TEA in 40% ACN, 80 °C	UV	Purity—degradation RNA products, short fragments and aggregates	Stability indicating method (direct exposure to heat, hydrolytic conditions, and treatment with ribonucleases).	[50]
- EPO (859 nts) - β-FLuc (~2000 nts)	IP-RP	ACQUITY premier oligonucleotide BEH C18 (300 Å, 1.7 μm, 2.1 × 150 m)	MPA: 0.1 M OAA, 1% HFIP in 40% ACN MPB: 0.1 M OAA, 1% HFIP in 90% ACN	UV	Poly(A) tail length	DIPEA/HFIP is a suitable ion-pairing system for sensitive LC MS analysis.	[10]
- eGFP (996 nts) - FLuc (1909 nts) - β-Gal (3420 nts)	IP-RP	DNAPac RP (150 mm × 2.1 mm i.d.)	MPA: 50 mM TEA, 50 mM HFIP in H_2_O MPB: 25 mM TEA, 25 mM HFIP in 90% MeOH. 80 °C	UV	Purity—short fragments and aggregates (5′loopback dsRNA)	Aggregates observed in IP-RP are associated with 5′loopback dsRNA impurities.	[30]
- zsGreen (~1000 nts) - mScarlet (~1000 nts)	IP-RP	DNAPac RP (150 mm × 2.1 mm i.d.)	MPA: 50 mM TEA, 50 mM HFIP in H_2_O Mobile phase B: 25 mM TEA, 25 mM HFIP in 90% MeOH. 80 °C	UV	Purity—short fragments	The use of different T7 polymerase directly impacted the level of short mRNA fragments.	[51]
Circular eGFP RNA	IP-RP	AcclaimTM 300 C18 (3 μm, 150 × 4.6 mm)	MPA: 100 mM TEAA in H_2_O MPB: 5% 100 mM TEAA in 95% ACN	UV	Purity, impurity analysis	Identification of circRNAs and nicked RNAs, and elucidated the degradation pattern of the circRNA substance.	[52]

AEX, anion exchange; ACN, acetonitrile; circRNA, circular RNA; DIPEA, N,N-di isopropyl-ethylamine; HFIP, 1,1,1,3,3,3,-hexafluoro-2-propanol; IP-RP, ion-pairing reversed phase; MALS, multi-angle light scattering detection; MeOH, methanol; MgCl, magnesium chloride; MPA, mobile phase A; MPB, mobile phase B; MS, mass spectrometer; MW, molecular weight; NaOH, sodium hydroxide; NTPs, nucleoside triphosphates; OAA, octyl ammonium acetate; OH-PEG, hydroxy PEG; Tris-HCl, tris(hydroxymethyl)aminomethane hydrochloride; RI, refractive index; SEC, size exclusion chromatography; TEA, triethylamine; TEAA, triethylammonium; TMAC, tetramethylammonium chloride.

**Table 4:** Summary of advanced sequencing methods for mRNA quality assessment.

Platform	Method/Application	Method Overview	Advantages	Limitations	Ref
Illumina	Sequence identity	Library preparation involves cDNA synthesis, fragmentation, adapter ligation, and PCR amplification	High-throughput, high-quality data	Length (standard < 300 bp; 600 bp max on MiSeq and NextSeq1000/2000 instruments)	[91]
	PAL-Seq: Poly(A) tail profiling	Labels poly(A) tails with biotinylated dUTP for fluorochrome detection	High throughput; effective poly(A) tail measurement	Requires outdated technology; limited by reliance on older sequencers	[92]
	TAIL-Seq: Poly(A) tail profiling	Uses fluorescence quantification to estimate poly(A) tail length	Compatible with modern sequencers; measures both the length of the poly(A) tails and their modifications, such as uridylation	Complex data analysis: requires tailored control software; intermediate fluorescence data storage; needs a substantial amount of RNA	[93]
	mTAIL-Seq: Poly(A) tail profiling	Modified TAIL-Seq with hairpin adapters for improved efficiency	Enhances the efficiency of ligation, reducing the amount of starting material needed, providing similar capabilities to those of TAIL-Seq	Shares the technical complexity and data analysis challenges of TAIL-Seq	[94]
	Poly(A)-Seq: Poly(A) tail profiling	Analyzes the lengths and internal compositions of poly(A) tails, uses oligo(dT) selection and 3′ adapter ligation	Can detect detailed compositions, including guanosine insertions within tails	May bias toward longer tails due to the selection method used, loss of information on tails shorter than ten nucleotides due to data filtering	[95]
	PAT-Seq: Poly(A) tail profiling	Extends RNA’s 3′ end with Klenow DNA polymerase	Can measure poly(A) tails and analyze changes in gene expression	Requires precise size selection, which can impact result accuracy; constrained by the length of sequencing reads, making it difficult to detect very long tails	[96]
	EnD-Seq: Non-Poly(A) tail analysis	Targets short poly(A) tails and non-polyadenylated RNAs and specializes in analyzing non-poly(A) modifications at RNA 3′ ends	Highly sensitive to short and non-poly(A) tails, useful for examining RNA decay products	Not suitable for assessing long poly(A) tails, specific improvements in sensitivity may affect the results	[97]
	3′RACE-Seq: Non-Poly(A) tail analysis	Analyzes specific transcripts’ 3′ ends	Enables detailed sequencing of particular RNA 3′ ends, can identify modifications at the 3′ end	Limited to specific RNA targets, nested PCR steps might introduce bias	[98]
	TED-Seq: 3′ end mapping	Maps 3′ cleavage and polyadenylation sites	Cost-effective; achieves 10 bp resolution; straightforward protocol	Lacks quantitative robustness; variable recovery rates; may require precise size selection	[99]
	Ribo-Seq: ribosome profiling, translation efficiency	Captures the positions of ribosomes on mRNA transcripts by sequencing ribosome-protected mRNA fragments	Can reveal translation efficiency and identify active reading frames providing insights into translation dynamics and protein synthesis	Complex sample preparation and data analysis, necessitating specialized expertise and resources, which might limit accessibility	[100,101]
Oxford Nanopore Technologies	VAX-seq	Used for plasmid sequencing as well as IVT-produced RNA with both direct RNA sequencing and cDNA sequencing	Comprehensive workflow developed to assess mRNA vaccine quality by analyzing sequence identity, integrity, and poly(A) tail length	Expertise in long-read sequencing and complex data analysis required	[102]
	nanoSHAPE: RNA structure	Chemical probing method to analyze RNA structure	Structural insights can inform the design and optimization of mRNA vaccines, potentially improving their stability and translation efficiency	Efficiency and specificity of the chemical modification can vary, potentially leading to incomplete or biased structural data if not carefully controlled	[103]
	Integrity analysis with Nano3P-seq	Captures both polyadenylated and non-polyadenylated RNA; uses a template-switching reverse transcriptase (TGIRT) to capture the full-length RNA	Long reads that can cover entire RNA molecules, including poly(A) tails to enable broad RNA analysis with no PCR bias	Higher error rate compared with other sequencing technologies, requires more complex data analysis	[104]
	FLEP-seq	Captures both polyadenylated and non-polyadenylated RNA; adds DNA adapter at 3′ end and RT	Long reads that can cover entire RNA molecules, RNA polymerase II positioning, splicing status, polyadenylation sites, and poly(A) tail lengths and translation efficiency	Higher error rate compared with other sequencing technologies	[105]
	RNA modification detection	Direct RNA sequencing	Direct sequencing of RNA uses RNA adapters, no need for reverse transcription or amplification and multiplexing capabilities	Higher error rates compared with other sequencing technologies	[91,102]
PacBio	Sequence identity analysis, full-length mRNA sequencing	Library prep involves cDNA synthesis, adapter ligation, and circular consensus sequencing (CCS)	Long, high-fidelity/quality reads	High cost, PCR amplification bias, instrument generally less available than other platforms	[106]
	FLAM-Seq: Poly(A) tail analysis	Poly(A) selection followed by enzymatically added G/I tail serving as anchor for oligo prior to RT	Low error rate, long reads, to analyze full-length mRNA and their poly(A) tails—can detect internal variations within the tails	May introduce biases from PCR amplification steps, requires a significant amount of RNA material	[107]
	PAIso-Seq: Poly(A) tail analysis	3′-end extension of template oligo containing T stretch	Low error rates, long reads, to measure poly(A) tails without needing to pre-select polyadenylated RNA, can be performed with 100 ng of RNA or less	Potential issues with artifacts from the extension process	[108]
	SM-PAT Poly(A) tail analysis	Poly(A) selection followed by the addition of a 3′ adapter through splint ligation	Low error rates, long reads; very similar to FLAM-Seq and PAIso-Seq	May introduce biases through PCR amplification steps	[109]

**Table 5:** Summary of the advantages and limitations of each analytical technology.

Analytical Technology	Advantages	Limitations
Capillary gel electrophoresis	Effectively separate and provide information on abortive transcripts (i.e., truncated and degraded mRNA) and long 3′-loopback dsRNA byproducts. The miniaturized format ensures rapid analysis with comparable resolution.	Limited capability to detect 3′-heterogeneity transcripts and short 3′-loopback dsRNA byproducts because the size differences between these short species and the full-length mRNA are too small to be separated. Limited resolution for mRNA analysis over 4000 nts.
Anion-exchange chromatography	Enable the determination of mRNA purity and monitoring of IVT components.	Limited resolution for intact RNA analysis. Requires specific conditions, such as the use of denaturing conditions or ion-pairing agents, to achieve optimal separation. Sensitive to the presence of impurities and variations in the mobile phase, affecting reproducibility and accuracy of results.
Size exclusion chromatography	Provide information on the aggregate amount. Enable the determination of molecular weight when used in association with a MALS detector.	Limited pore size of conventional columns, resulting in poor separation. Ultrawide pore SEC columns have been introduced in order to improve the separation of mRNAs and lipid nanoparticles.
Ion-pair reversed phase liquid chromatography	Enable the determination of mRNA purity and especially the level of abortive transcripts and/or mRNAs with partial poly(A) tail. Compatible with mass spectrometry for characterization of structural features, such as poly(A) tail length, 5′ end capping efficiency, or sequencing after a digestion step.	The limit of resolution does not permit determination of the level of short 3′-extended species. Limited resolution for mRNA analysis over 4000 nts. When coupled with MS, LC-MS instrument needs to be dedicated to mRNA analysis due to the use of ion pairing agents.
Mass photometry	Applicable for large molecules (up to 5 MDa) and able to measure the level of sense-antisense dsRNA byproducts and aggregates (i.e., dimers and trimers).	Limited resolution for the separation of abortive transcripts and t 3′-loopback dsRNA byproducts.
Native mass spectrometry	Applicable theoretically to any size/complexity for mass analysis. Fast analysis, low amount of sample required, fast sample preparation. No HPLC column needed, and mRNA are analyzed under native conditions.	Costly and requiring MS expertise. Lack of LC separation can lead to non-detection of minor variants.
Sanger sequencing	Considered as the gold standard for mRNA sequence verification.	Labor-intensive and less effective for analyzing large or complex sequences. It involves challenging primer design and has limited sensitivity for detecting low-frequency variants or contaminants, making it less suitable than NGS for comprehensive mRNA quality assessments.
Illumina sequencing	High accuracy (99.9%). High throughput, cost-effective for bulk RNA-seq. Well-established analysis pipelines.	Short reads (50–300 bp) limit isoform detection. Struggles with full-length mRNA and poly(A) tail analysis.
PacBio sequencing	High accuracy (~99.9%) with HiFi reads. Full-length mRNA isoform resolution. Detects splice variants and structural variants. Lower throughput and higher cost per base. Requires high input RNA amounts.	Lower throughput and higher cost per base. Requires high input RNA amounts.
Oxford nanopore	Direct RNA sequencing (no PCR bias). Detects poly(A) tail length, base modifications, and full-length transcripts. Portable and real-time sequencing.	Lower accuracy (~90–98%), prone to indels (especially deletions). Requires optimization for error correction.
